# Synthesis and biological evaluation of cleistocaltone A, an inhibitor of respiratory syncytial virus (RSV)†

**DOI:** 10.1039/d4sc01897d

**Published:** 2024-05-24

**Authors:** Lorenz Wiese, Sophie M. Kolbe, Manuela Weber, Martin Ludlow, Mathias Christmann

**Affiliations:** a Institute of Chemistry and Biochemistry, Freie Universität Berlin 14195 Berlin Germany mathias.christmann@fu-berlin.de; b Research Center for Emerging Infections and Zoonoses, University of Veterinary Medicine Hannover Foundation 30559 Hanover Germany

## Abstract

The first chemical synthesis of the phloroglucinol meroterpenoid cleistocaltone A (1) is presented. This compound, previously isolated from *Cleistocalyx operculatus* was reported to show promising antiviral properties. Based on a modified biosynthesis proposal, a synthetic strategy was devised featuring an intramolecular Diels–Alder reaction and an epoxidation/elimination sequence to generate the allyl alcohol handle in the side chain. The strategy was successfully executed and synthetic cleistcaltone A was evaluated against a contemporary RSV-A strain.

## Abstract 2

The first chemical synthesis of the phloroglucinol meroterpenoid cleistocaltone A (1) is presented.

## Graphical abstract

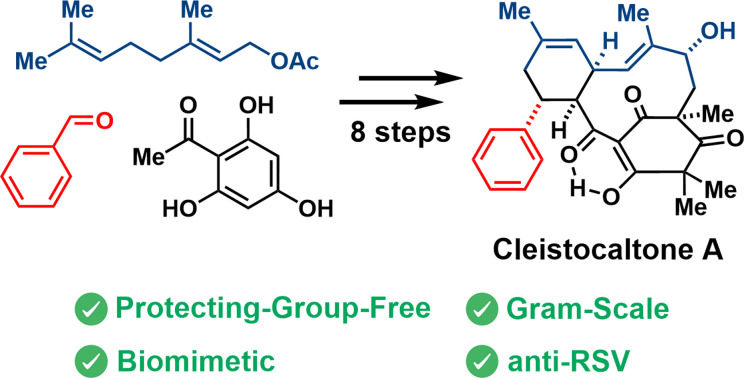

## Introduction

**Fig. 1:**
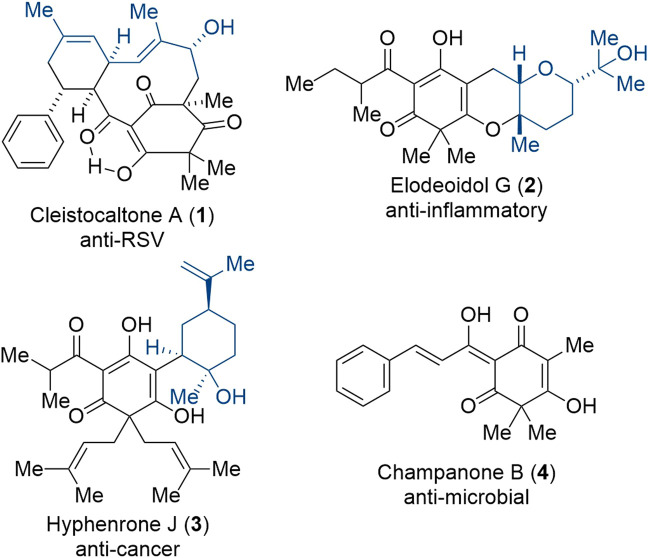
Example for different phloroglucinol meroterpenoids with various biological activities.^12,14–16^

Viral infections pose a significant economic burden and societal threat worldwide.^1–3^ The recent COVID-19 pandemic caused by severe acute respiratory syndrome coronavirus 2 (SARS-CoV-2) has starkly highlighted the risks posed by respiratory viruses to vulnerable populations and the urgent need for effective antivirals. Respiratory Syncytial Virus (RSV) is also a potent respiratory pathogen and is responsible for high levels of acute lower respiratory tract infections in infants, the elderly and immunocompromised adults.^4,5^ Despite the urgency, the current landscape of antiviral treatments is sparse, underscoring the critical need for further antiviral development.^6^ Natural products have a long and successful history as privileged starting points in the search for novel therapeutic approaches to counter infectious diseases.^7–9^ One effective strategy for navigating their vast chemical space is to deconvolute molecular compositions of herbal remedies described in traditional Chinese medicine (TCM), thus building upon a wealth of knowledge based on centuries of use.^10^ For example, the genus *Cleistocalyx*, belonging to the *Myrtaceae* family, comprises various plant species, some of which are used in Southeast Asia to create medicinal teas. *Cleistocalyx operculatus* (Roxb.) Merr. and Perry, a tree native to southern China, has been traditionally used to treat cold, fever, and inflammation with extracts from its buds and leaves.^11^ Cleistocaltone A (1, Fig. 1), a polymethylated phloroglucinol meroterpenoid (PPM) isolated from the buds of *C. operculatus* by Ye, Wang, and co-workers in 2019, was demonstrated to possess promising *in vitro* activity against RSV.^12^

In nature, components from diverse classes of natural products frequently merge to form compounds with novel biological activities. Among these classes, phloroglucinol meroterpenoids stand out.^13^ These compounds consist of a phloroglucinol segment and a terpene segment. The terpene portion typically originates from a geranyl group, which undergoes a series of biosynthetic transformations involving oxidation and cyclization. For instance, elodeoidol G (2, Fig. 1), an anti-inflammatory agent isolated by Luo, Kong, and coworkers, features a terpene moiety proposed to be biosynthesized from an oxidized geranyl building block through a series of epoxidations and an acid-catalyzed epoxide opening cascade.^14^ Similarly, the cytotoxic compound hyphenrone J (3, Fig. 1), isolated by Qin, Xu, and coworkers, contains two prenyl groups and an oxidized geranyl side chain.^15^

**Scheme 1:**
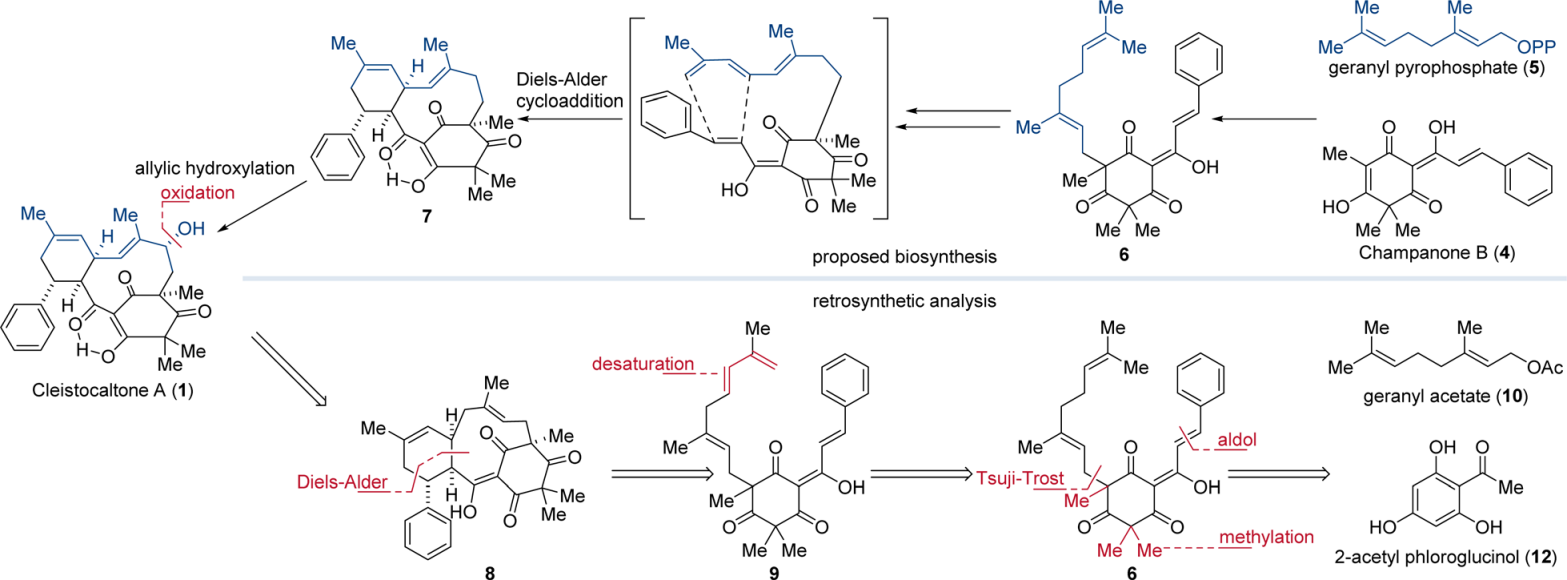
Biosynthesis of cleistocaltone A (1) proposed by Ye, Wang, and coworkers and retrosynthetic analysis both featuring an IMDA as the key step.^12^

Biosynthetically, the phloroglucinol portion of cleistocaltone A (1) is proposed to originate from champanone B (4, Fig. 1). Addition of geranyl pyrophosphate (GPP, 5) then forms geranyl champanone B 6. Subsequent desaturation of the geranyl terminus and isomerization of the homobenzylic double bond generates a triene, enabling an intramolecular Diels–Alder cycloaddition (IMDA) with the cinnamoyl dienophile (Scheme 1).

This reaction forges the characteristic 10-membered macrocycle found in 7 (Scheme 1).^12^ Ye, Wang, and coworkers succeeded in isolating 24 mg of (±)-cleistocaltone A (1) from 15 kg of plant material, highlighting the scarcity of this compound from biological sources.^12^ This underlines the significance of chemical synthesis to produce substantial quantities of (±)-cleistocaltone A (1) for further biological evaluation.

In recent years, biomimetic strategies have emerged as an intriguing and efficient approach for synthesizing complex natural products. These strategies elegantly mimic the pathways found in nature.^17–19^ By harnessing the innate reactivity of molecules, biomimetic syntheses often minimize the need for protection groups, resulting in greater efficiency and sustainability.^20^

Mirroring the proposed biosynthetic pathway, we envisioned an IMDA as the pivotal step for constructing the 10-membered macrocycle of 8. To generate the diene moiety in 9 for the IMDA, we planned a selective desaturation at the terminus of the geranyl group in 6, obtained from geranyl acetate (10). This geranyl unit would be attached to the nucleophilic carbon atom of methylated phloroglucinol 11 using a Tsuji–Trost alkylation.^21^ The phloroglucinol building block would be synthesized from commercially available 2-acetyl phloroglucinol (12) through trimethylation and an aldol condensation.

## Results and discussion

**Scheme 2:**
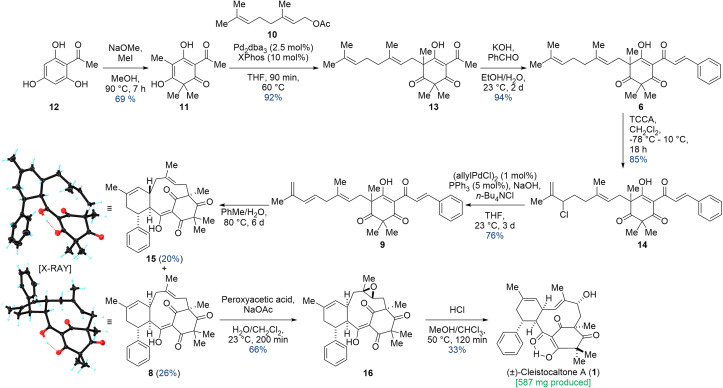
Linear synthesis of cleistocaltone A (1) starting from 2-acetyl phloroglucinol (12) and geranyl acetate (10).

Our synthesis, as outlined in Scheme 2, commenced with the literature-known trimethylation of 2-acetyl phloroglucinol (12), employing sodium methoxide and methyl iodide.^22^

Following the neutralization of the reaction mixture, the desired product precipitated, allowing for straightforward separation *via* filtration. This workup efficiently removed impurities and byproducts, including dimethylated 2-acetyl phloroglucinol, which stayed in solution. Consequently, we obtained trimethylated acetyl phloroglucinol 11 with a yield of 69%. The obtained trimethylated acetyl phloroglucinol 11 was then coupled with commercially available geranyl acetate (10) in a Tsuji–Trost allylic alkylation. Using Pd_2_dba_3_ as a Pd(0) source and testing multiple ligands, XPhos proved superior, yielding the geranylated phloroglucinol 13 as a mixture of interconverting tautomers with an excellent yield of 92%. Rendering the coupling reaction enantioselective would be highly desirable. The single stereocenter formed in the Tsuji–Trost reaction ultimately dictates whether (+)- or (−)-cleistocaltone A (1) is synthesized due to the diastereoselectivity of subsequent steps. Unfortunately, despite rigorous screening, we could not identify a chiral ligand that induced significant enantiomeric excess. Consequently, we proceeded with the synthesis using a racemic mixture of geranylated acetyl phloroglucinol 13.

In the subsequent aldol condensation, 13 was reacted with benzaldehyde under basic conditions. Due to a competing Cannizzaro reaction consuming benzaldehyde (yielding benzyl alcohol and benzoic acid),^23^ a portion-wise addition of 6 equivalents of aldehyde was crucial for complete conversion. Then, the high solubility of 6 in cyclohexane was exploited for extraction, leaving benzoic acid and much of the benzyl alcohol in the aqueous phase. Unreacted benzaldehyde was removed under high vacuum, greatly streamlining the purification process. This yielded geranyl champanone B 6 as a mixture of tautomers in 94% yield.

The geranyl moiety in compound 6 was selectively chlorinated at its terminus using trichloroisocyanuric acid (TCCA) in CH_2_Cl_2_. This double bond exhibited the highest reactivity presumably due to a lack of negative steric (homoallylic quaternary center) and electronic influences (cinnamoyl group). Careful temperature control was crucial for this reaction. While the reaction proceeded smoothly on a milligram scale (85% yield of 14, simple filtration), scaling up to gram quantities significantly decreased the yield to 50% and complicated the column chromatography purification due to increased side-product formation. 14 was isolated as a diastereomeric mixture each consisting of interconverting tautomers. In the subsequent step, the allylic chloride in 14 was eliminated *via* a palladium-catalyzed β-hydride elimination to afford diene 9 as a mixture of two interconverting tautomers in 76% yield. The combination of sodium hydroxide and the phase-transfer catalyst tetrabutylammonium chloride ensured efficient removal the liberated hydrochloric acid (HCl).^24^

With the required structural elements assembled, a thermal IMDA was performed in a biphasic mixture of water and toluene. This process afforded diastereomers 15 and 8 in 20% and 26% yields, respectively. Crystallization proved to be the most efficient purification method. Treatment of the crude product with pentane followed by cyclohexane preferentially dissolved the undesired IMDA diastereomer 15, leaving the desired diastereomer 8 as a solid. While this purification was extremely convenient, some amount of 8 also dissolved, resulting in a reduced yield of only 26% (compared to a possible 32% as determined by ^1^H-NMR). The relative configurations of 8 and 15 were confirmed by XRD analysis.

In exploring the impact of various solvents on the IMDA reaction's diastereomeric ratio, a 3 : 1 excess of the undesired diastereomer 15 was observed in both pyridine and *n*-butanol. Unfortunately, no other tested solvent yielded an excess of the desired diastereomer 8. Consequently, a toluene/water mixture was chosen, allowing the reaction to proceed at a lower temperature (80 °C), which minimized side product formation and simplified purification.

The epoxidation of 8 presented a challenge, as its two trisubstituted double bonds appeared electronically similar. We speculated that strain-release in the transition state would render the macrocyclic double bond significantly more reactive. The facial preference was predicted from the X-ray crystal structure of the starting material. Treatment of 8 with peroxyacetic acid and sodium acetate in a biphasic water/dichloromethane mixture selectively furnished epoxide 16 as a single diastereomer in 66% yield.

**Scheme 3:**
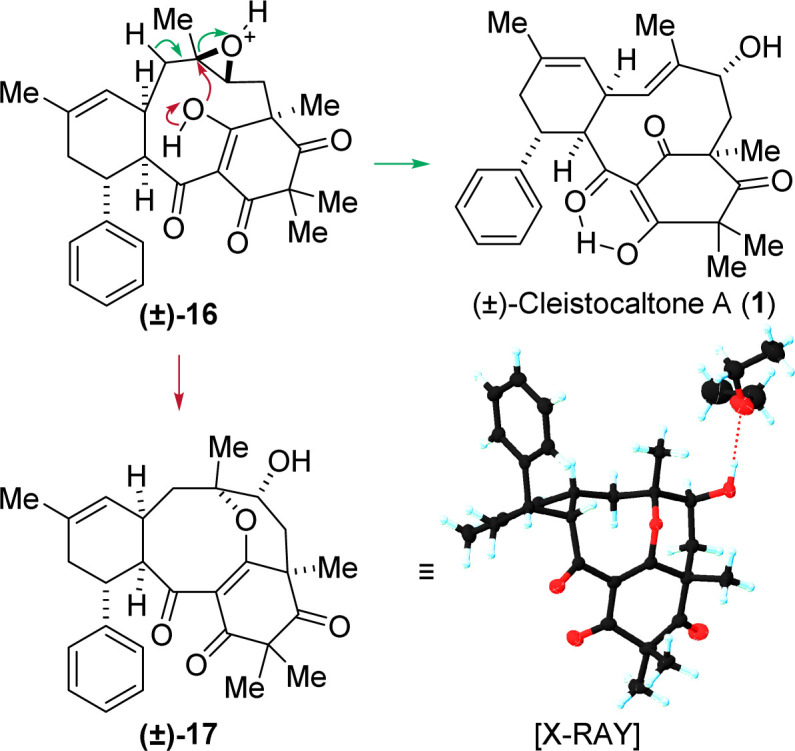
Proposed mechanism for the formation of (±)-cleistocaltone A (1) *via* an acid mediated epoxide opening and cyclic ether (±)-17*via* a nucleophilic attack of the enol-OH in epoxide (±)-16. Racemic 17 crystallizes as a conglomerate. The configuration of the enantiomer present in the obtained crystal was opposite to that shown for (±)-17.

Various conditions for opening epoxide 16 were investigated. Lewis acidic conditions generally led to decomposition. Basic conditions, even using strong bases such as *n*-butyl lithium at room temperature, resulted in no conversion. This lack of reactivity is attributed to steric hindrance, with the macrocycle shielding the acidic α-position adjacent to the epoxide. Fortunately, Brønsted acidic conditions proved successful. Treatment with HCl at 50 °C opened the epoxide, yielding the allylic alcohol and, ultimately, cleistocaltone A (1) in a 33% yield. Additionally, the acid-mediated opening of the epoxide in 16 resulted in an intriguing side product 17, characterized by an unusual tetracyclic structure that includes an eight-membered cyclic ether. The structure was confirmed through XRD analysis. It is proposed that this product originates from a nucleophilic attack by the enol-oxygen on the tertiary carbon center, a process initiated by the protonation of the epoxide (Scheme 3).

**Scheme 4:**
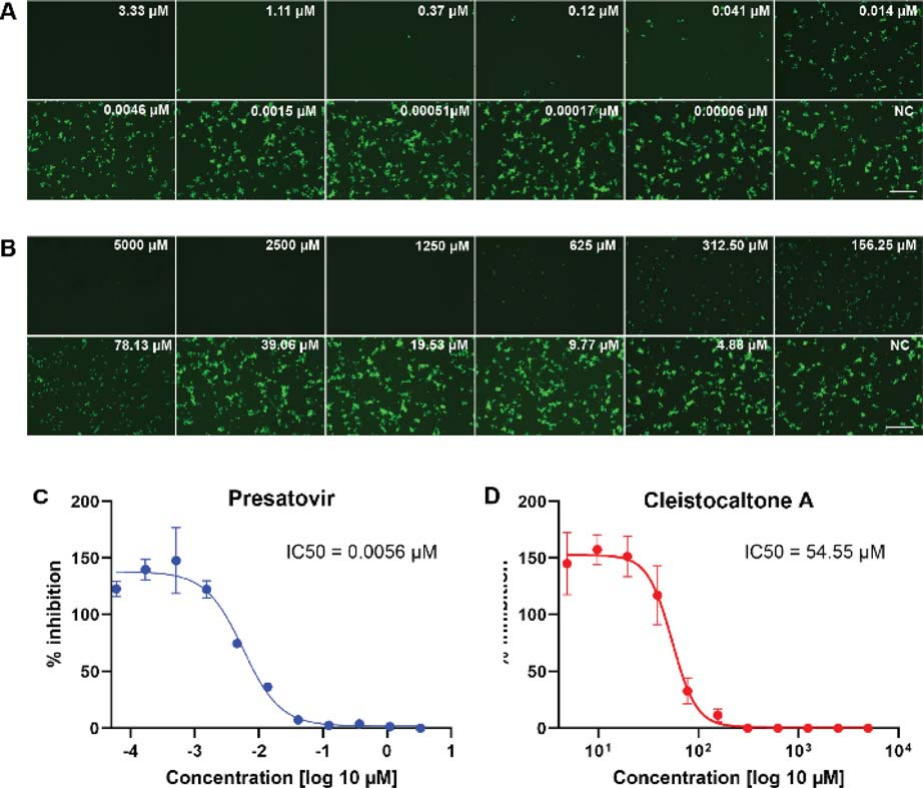
Assessment of the antiviral efficacy of cleistocaltone A (1) against RSV. (A and B) Vero cells were pretreated with three-fold serial dilutions of the fusion inhibitor Presatovir or two-fold serial dilutions of cleistocaltone A (1) and infected with rRSV-A-0594-EGFP (2000 TCID_50_/well) in the presence of each compound. Fluorescence photomicrographs showing EGFP fluorescence were obtained by UV microscopy at 48 hours post-infection. Scale bars, 500 μm. (C and D) Quantification of EGFP fluorescence in drug treated Vero cells was performed in 4% PFA fixed cells at 48 h.p.i. using a Tecan Infinite 2000 plate reader and normalized to untreated controls wells containing 0.1% DMSO. IC_50_ values were determined by nonlinear regression analysis using GraphPad Prism 10. Error bars represent the standard deviations from two independent experiments (*n* = 8).

We investigated the antiviral efficacy of synthetic cleistocaltone A (1) against a contemporary recombinant RSV-A strain (0594, genotype ON1)^25^ using an *in vitro* model system (Scheme 4). Presatovir, a known selective and potent inhibitor of RSV-induced cell fusion,^26^ served as a positive control. We confirmed Presatovir's high antiviral efficacy in infected Vero cells, with an IC_50_ of 0.0056 μM. Cleistocaltone A (1) also inhibited RSV infection, with a modestly higher IC_50_ of 54.55 μM. Although the IC_50_ of synthetic (±)-cleistocaltone A (1) is about tenfold higher than that reported for the isolated racemic compound (IC_50_ = 6.75 ± 0.75 μM),^12^ it remains within a relevant range.

## Conclusion and outlook

We successfully executed a biomimetic gram-scale synthesis of cleistocaltone A (1) using an efficient eight-step route with an overall yield of 2.2%. Our approach enables further derivatization at various stages to obtain synthetic analogues for structure–activity-relationship (SAR) studies, paving the way for potential drug development.

## Biological samples

Vero and HEp-2 cells were obtained from the American Type Culture Collection (ATCC, Manassas, USA).

## Data availability

All data (experimental procedures and characterization) that support the fndings of this study are available within the article and its ESI.† Crystallographic data for compounds 8, 15, and 17 have been deposited with the Cambridge Crystallographic Data Centre under CCDC2352922, CCDC2352924, and CCDC2352923.

## Author contributions

M. C. supervised the project. L. W. developed the synthesis and carried out all synthetic experiments. M. L. and S. K. developed and conducted all biological testing. M. W. performed all XRD measurements. L. W., M. C., and M. L. wrote the manuscript.

## Conflicts of interest

There are no conflicts to declare.

## Supplementary Material

SC-015-D4SC01897D-s001
