# Supplementary material for: Synthesis and biological evaluation of cleistocaltone A, an inhibitor of respiratory syncytial virus (RSV)
Source: Chem Sci. 2024 May 24;15(26):10121–5. doi: 10.1039/d4sc01897d (PMC11220581; doi:10.1039/d4sc01897d)
Supplement: SC-015-D4SC01897D-s001 [file SC-015-D4SC01897D-s001.pdf]

Electronic Supplementary Material (ESI) for Chemical Science.
This journal is © The Royal Society of Chemistry 2024

Synthesis and Biological Evaluation of Cleistocaltone A

Lorenz Wiese,[a] Sophie Kolbe,[b] Manuela Weber,[a] Martin Ludlow*[b] and Mathias Christmann*[a]

[a]Institute of Chemistry and Biochemistry, Freie Universität Berlin, 14195 Berlin, Germany.

[b]Research Center for Emerging Infections and Zoonoses, University of Veterinary Medicine Hannover Foundation, 30559 Hanover, Germany.

*E-Mail: m.christmann@fu-berlin.de

Supporting Information

Table of Contents

1. General Information **1**

1.1 Materials and Methods **1**

1.2 Analysis **1**

1.3 Antiviral Inhibition Assay **2**

2. Scheme S1: Linear Route to Intermediate 9 **3**

3. Scheme S2: Convergent Route to Intermediate 9 **3**

4. Scheme S3: IMDA and Backbone Oxidation **4**

5. Table S1: Ligand Screening for the Enantioselective Tsuji-Trost Coupling of Geranyl Acetate (10) and Trimethylated Phloroglucinol 11 **4**

6. Figure S1: Structures of Chiral Ligands from Solvias Asymmetric Screening Kit[6] **5**

7. Table S2: Solvent Screening for the IMDA **6**

8. Experimental Procedures and Analytical Data **7**

8.1 Linear Route to Intermediate 9 **7**

8.2 Convergent Route to Intermediate 9 **13**

8.3 IMDA and Backbone Oxidation **17**

9. Comparison of NMR Data **22**

9.1 (±)-Cleistocaltone A (1) **22**

10. X-ray data **23**

10.1 IMDA-diastereomer 15 (CCDC2352924) **23**

10.2 IMDA-diastereomer 8 (CCDC2352922) **24**

10.3 Side Product 17 (CCDC2352923) **25**

11. NMR Spectra **26**

12. References **39**

1. General Information

1.1 Materials and Methods

Reactions with air or moisture sensitive substances were carried out under an argon atmosphere using standard Schlenk technique. Ambient or room temperature (RT) refers to 18–23 °C. Heating of reactions was performed with an oil bath unless otherwise noted. “Brine” refers to a sat. aq. NaCl solution.

Unless otherwise noted, all starting materials and reagents were purchased from commercial distributors and used without further purification. Anhydrous dichloromethane and tetrahydrofuran were provided by purification with a MBraun SPS-800 solvent system (BRAUN) using solvents of HPLC grade purchased from FISHER Scientific and ROTH. 2-Acetyl phloroglucinol monohydrate was dried for 16 h at 60 °C in the high vacuum to obtain dry 2-acetyl phloroglucinol which was stored under argon. Solvents for extraction, crystallization and flash column chromatography were purchased in technical grade and distilled under reduced pressure prior to use.

Column chromatography was performed on silica 60 M (0.040-0.063 mm, 230-400 mesh, MACHEREY-NAGEL).

Medium pressure liquid chromatography (MPLC) was performed with a TELEDYNE ISCO Combi-Flash Rf200 using prepacked silica columns and cartridges from TELDYNE. UV response was monitored at 254 nm and 280 nm. As eluents, cyclohexane (99.5+% quality) and EtOAc (HPLC grade) were used.

1.2 Analysis

Reaction monitoring: Reactions were monitored by thin layer chromatography (TLC). TLC-analysis was performed on silica gel coated aluminium plates ALUGRAM® Xtra SIL G/UV_{254} purchased from MACHEREY-NAGEL. Products were visualized by UV light at 254 nm and by using staining reagents (based on $KMnO_4$, $Ce(SO_4)_2$ and anisaldehyde).

NMR spectroscopy: ^{1}H NMR and ^{13}C NMR spectral data were recorded on JEOL (ECX 400, ECP 500), VARIAN (Inova 600) and BRUKER (AVANCE III 500, AVANCE III 700) spectrometers in the reported deuterated solvents. The chemical shifts (δ) are listed in parts per million (ppm) and are reported relative to the corresponding residual non-deuterated solvent signal ($CDCl_3$: δ_H = 7.26 ppm, δ_C = 77.2 ppm; CD_2Cl_2: δ_H = 5.32 ppm, δ_C = 53.8 ppm; DMSO-d_6: δ_H = 2.50 ppm, δ_C = 39.5 ppm). Integrals are in accordance with assignments; coupling

constants (J) are given in Hz. Multiplicity is indicated as follows: s (singlet), d (doublet), t (triplet), q (quartet), p (pentet), br (broad) and combinations thereof. In the case where no multiplicity could be identified, the chemical shift range of the signal is given as m (multiplet). ^{13}C NMR spectra are ^{1}H-broadband decoupled.

High resolution mass spectrometry: High resolution mass spectra (HRMS) were measured with an AGILENT 6210 ESI-TOF (10 µL/min, 1.0 bar, 4 kV) instrument.

Infrared spectroscopy: Infrared (IR) spectra were measured on a Jasco FT/IR-4100 Type A spectrometer with a TGS detector. Wavenumbers $\tilde{\nu}$ are given in cm^{-1}.

X-ray: X-ray diffraction data was collected on a BRUKER D8 Venture CMOS area detector (Photon 100) diffractometer with Cu Kα radiation. Single crystals were coated with perfluoroether oil and mounted on a 0.2 mm Micromount. The structures were solved with the ShelXT[1] structure solution program using intrinsic phasing and refined with the ShelXL[2] refinement package using least squares on weighted F2 values for all reflections using OLEX2.[3]

1.3 Antiviral Inhibition Assay

Vero and HEp-2 cells were obtained from the American Type Culture Collection (ATCC, Manassas, USA) and cultured in Dulbecco's modified Eagle's medium (Thermo Fisher Scientific) supplemented with 10% fetal bovine serum (FBS), 1% penicillin/streptomycin, and 1% GlutaMAX and were cultured at 37 °C with 5% CO_2. Working stocks of rRSV-A-0594-EGFP, a recombinant respiratory syncytial virus (RSV) strain based on a contemporary subtype A strain, were generated, and titrated on HEp-2 cells as previously described.[4] Presatovir (GS-5806) was purchased from MedChemExpress and has been previously shown to act as an RSV fusion inhibitor.[5] Stock solutions of Cleistocaltone A (10 mM) and Presatovir (1 mM) were prepared in dimethyl sulfoxide (DMSO) (Sigma Aldrich), aliquoted and stored at -20 °C and -80 °C respectively. Vero cells grown in 96 well trays were pre-treated with either three-fold serial dilutions of Cleistocaltone A (**1**) or two-fold serial dilutions of Presatovir in Opti-MEM (Thermo Fisher Scientific) prior to infection with 2000 $TCID_{50}$/well of rRSV-A-0594-EGFP. Cells were incubated at 37 °C for 48 hours and inhibition of infection was assessed by visualization of EGFP fluorescence using UV microscopy. Cells were fixed in 4% (w/v) paraformaldehyde and the fluorescence quantified using a Tecan Infinite 2000 plate reader. The resulting values were normalized to rRSV-A-0594-EGFP infected Vero cells containing 0.1% dimethyl sulfoxide DMSO and are shown as the mean (n=8) of two biological replicates. IC_{50} values were determined by nonlinear regression analysis using GraphPad Prism 10.

2. Scheme S1: Linear Route to Intermediate 9

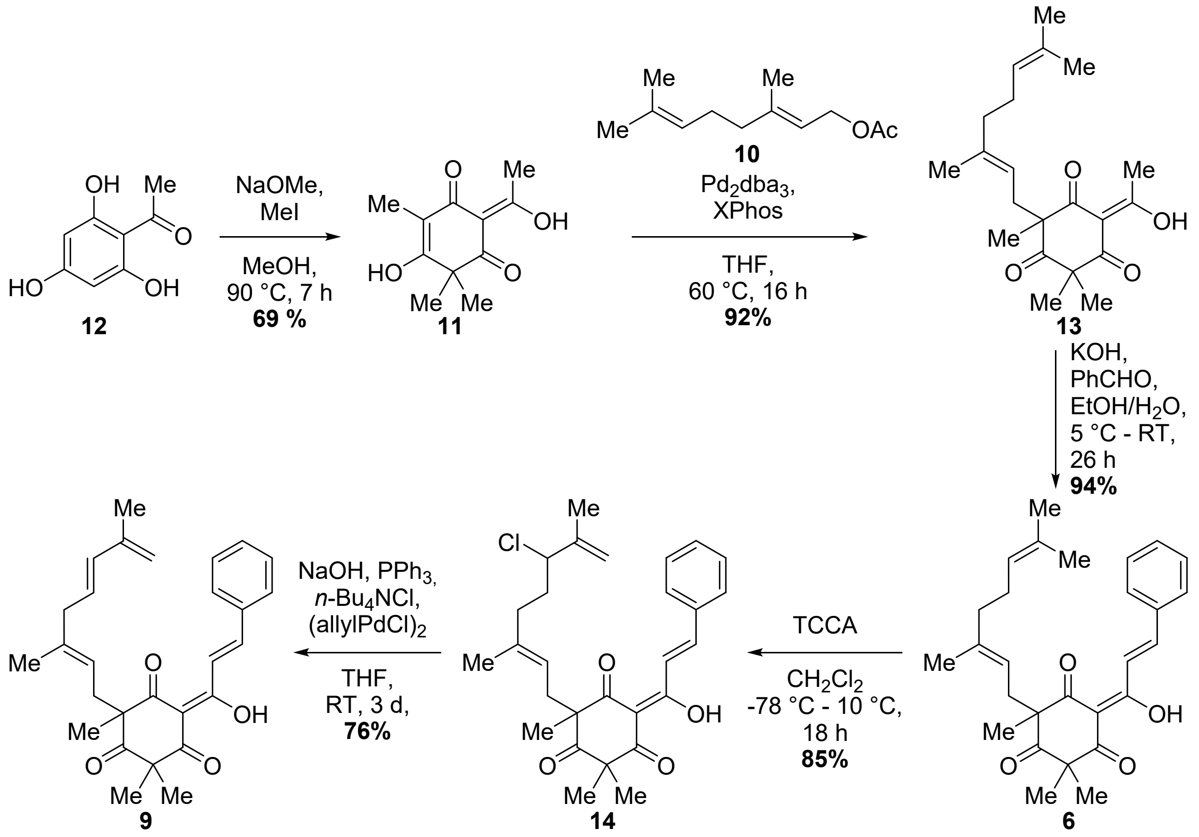

3. Scheme S2: Convergent Route to Intermediate 9

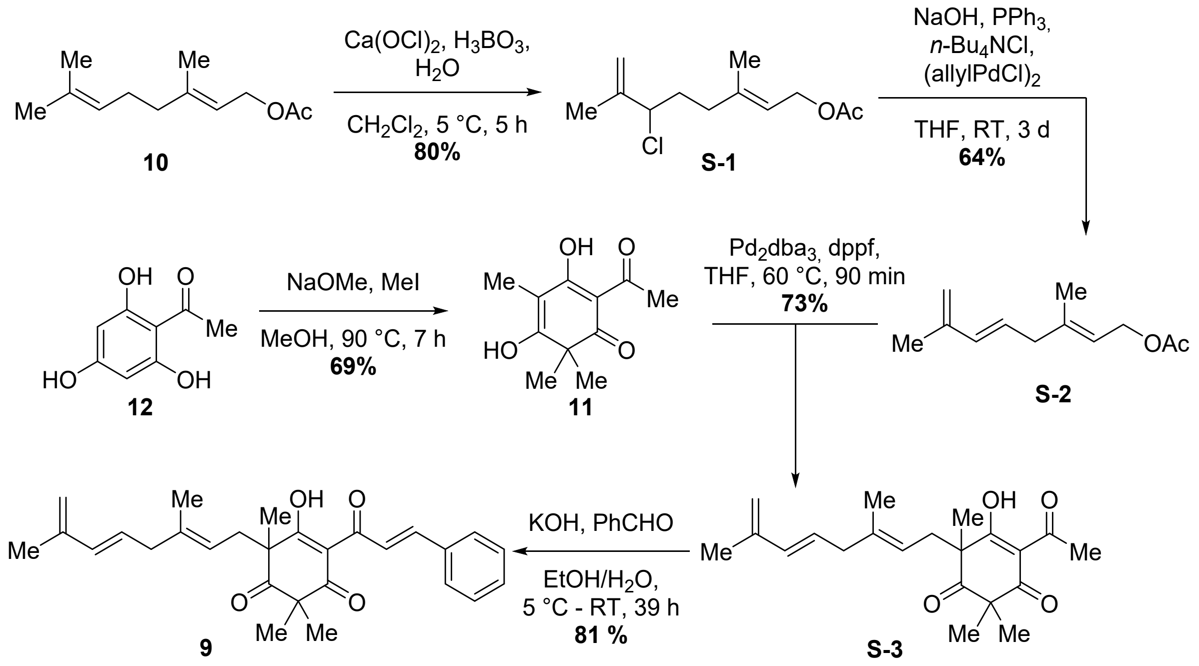

4. Scheme S3: IMDA and Backbone Oxidation

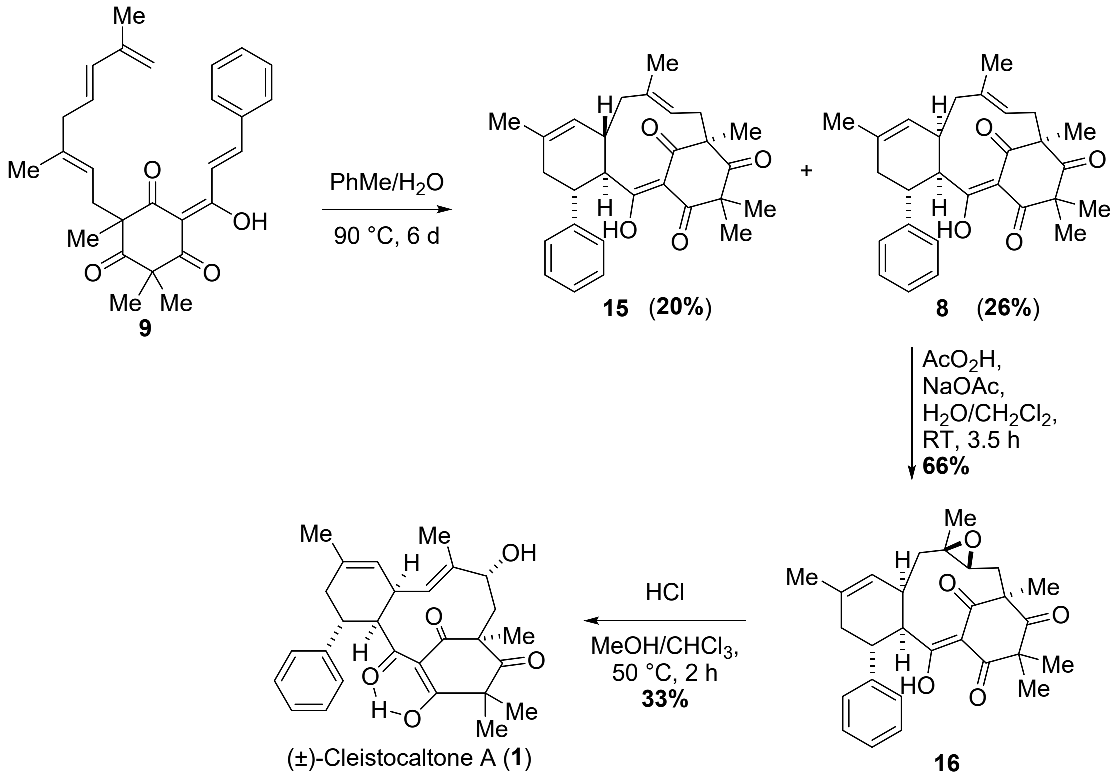

5. Table S1: Ligand Screening for the Enantioselective Tsuji-Trost Coupling of Geranyl Acetate (10) and Trimethylated Phloroglucinol 11

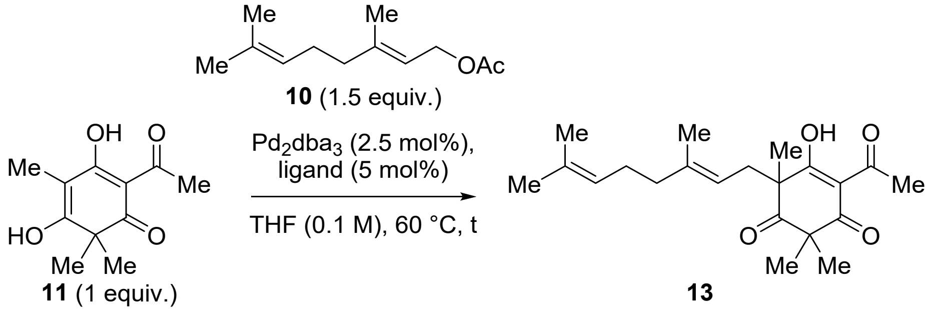

Entry	Ligand[a]	t (h)	Yield	*ee*[b]	Comment
1	(*R*)-BINAP	48	-	-	no conversion
2	DuPhos	48	-	-	no conversion
3	(*R*, *R*)-DACH-Trost-Ligand	36	22%	0%	
4	Josiphos-SL-J001-1	16	-	-	no conversion
5	Josiphos-SL-J002-1	16	-	-	no conversion
6	Josiphos-SL-J003-1	16	-	-	no conversion

7	Josiphos-SL-J005-1	16	-	-	no conversion
8	Taniaphos-SL-T001-1	16	-	-	no conversion
9	Taniaphos-SL-T002-1	16	-	-	no conversion
10	Walphos-SL-W001-1	16	12%	0%	-
11	Walphos-SL-W002-1	16	55%	0%	-
12	Mandyphos-SL-M001-1	16	61%	0%	-
13	Mandyphos-SL-M004-1	16	44%	0%	-
14	Rophos-SL-P001-2	16	-	-	no conversion
15	MeOBIPHEP-SL-A-101-1	16	-	-	no conversion
16	MeOBIPHEP-SL-A-109-1	16	-	-	no conversion

a) All ligands except for (*R*)-BINAP, Duphos and (*R*, *R*)-DACH-Trost-Ligand were used from Solvias Asymmetric Ligands Screening Kit.[6] b) All *ee* were determined by NP-HPLC using a Chiralpak IC column. The eluent consisted of 0.5% *iso*-propanol in *n*-hexane with a flow rate of 0.5 mL/min. The retention times for both enantiomers were 10.7 min and 11.5 min, respectively.

6. Figure S1: Structures of Chiral Ligands from Solvias Asymmetric Screening Kit[6]

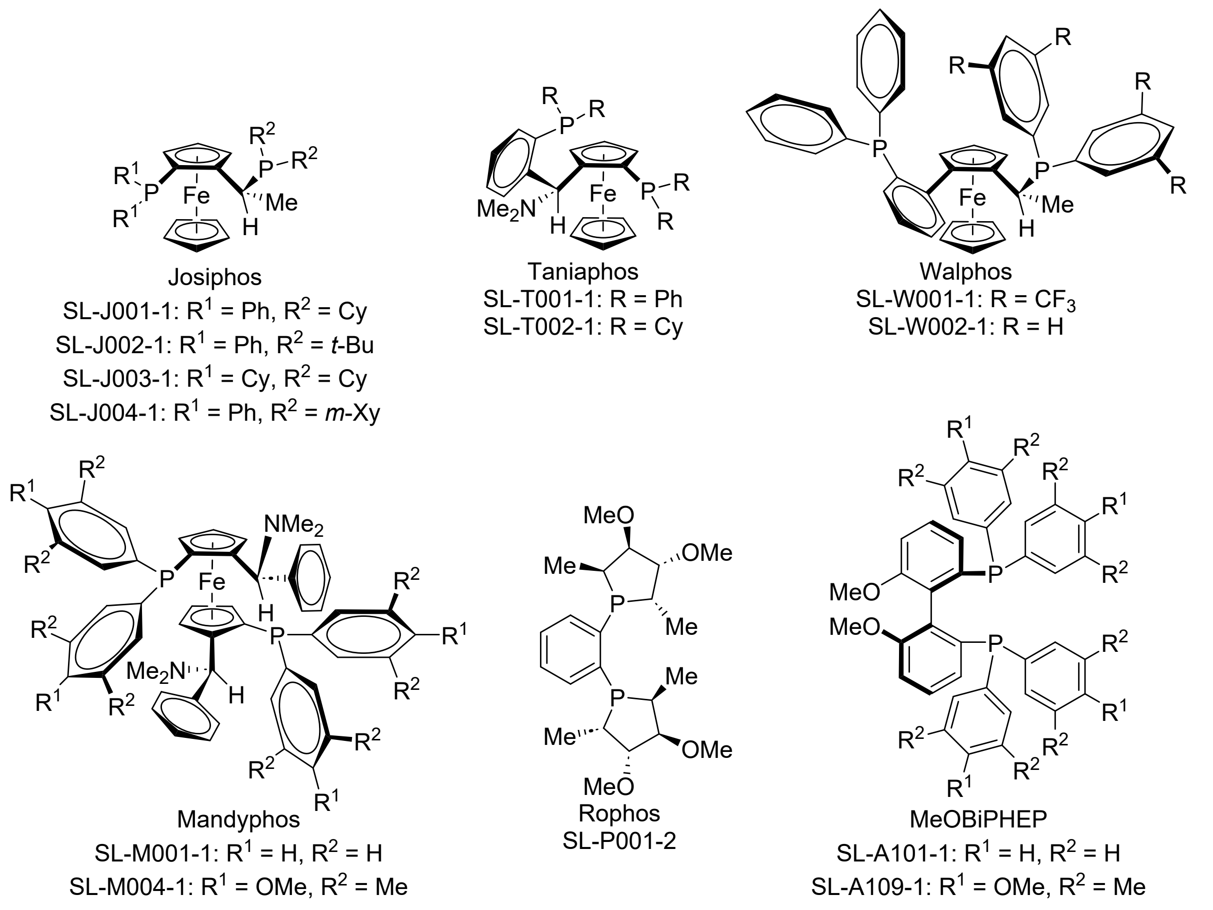

7. Table S2: Solvent Screening for the IMDA

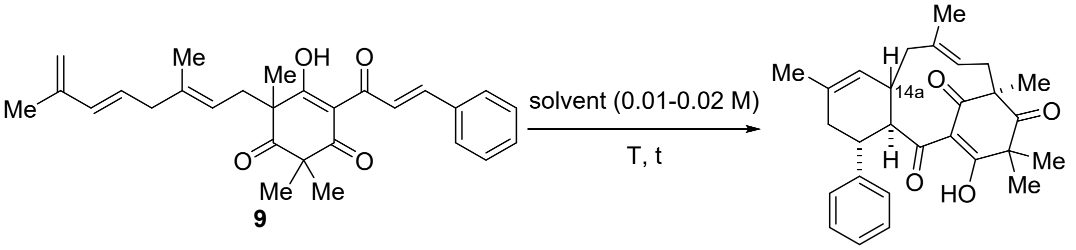

Entry	Solvent	t (d)	T (°C)	*dr* (15:8)[a]	Comment
1[b]	ethanol/water	4	110	5:2	-
2[b]	*n*-butanol	4	110	3:1	-
3[b]	*tert*-butanol	4	110	5:4	-
4[b]	pyridine	5	120	3:1	significant decomposition
5[b]	tetrahydrofuran	1	110	5:4	significant decomposition
6[b]	methyltetrahydrofuran	1	110	4:3	significant decomposition
7[b]	1,4-dioxane	5	120	5:4	-
8[b]	1,2-dimethoxyethane	1	110	4:3	-
9[b]	diglyme	5	120	4:3	-
10[b]	1,2-dichloroethane	1	110	4:3	-
11[b]	benzotrifluoride	5	120	10:7	-
12[b]	chlorobenzene	5	120	5:3	-
13[b]	toluene	5	110	10:7	-
14[b]	heptane	5	120	10:7	-
15	toluene/water	5	90	3:2	very facile purification

a) All *dr* were determined by crude ^{1}H-NMR using the integrals of the enol-O–H at 17.50 ppm for **15** and 17.96 ppm for **8**. b) Reaction was carried out in a capped vial.

8. Experimental Procedures and Analytical Data

8.1 Linear Route to Intermediate 9

8.1.1 2-Acetyl-3,5-dihydroxy-4,6,6-trimethylcyclohexa-2,4-dien-1-one (11)

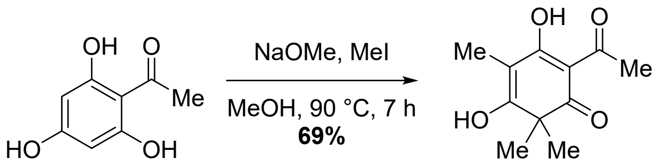

12 $C_8H_8O_4$ (168.15 g/mol) **11** $C_{11}H_{14}O_4$ (210.23 g/mol)

The trimethylation of 2-acetyl phloroglucinol (**12**) was performed according to a literature known procedure.[7] Sodium (7.59 g, 330 mmol, 3.7 equiv.) was added to anhydrous methanol (250 mL) in a three-necked round-bottom flask equipped with a reflux-condenser. After the sodium had reacted completely, 2-acetyl phloroglucinol (**12**, 15.0 g, 89.2 mmol, 1.0 equiv.) dissolved in anhydrous methanol (200 mL) was added slowly. Afterwards, methyl iodide (18.2 mL, 41.8 g, 294 mmol, 3.3 equiv.) was added slowly. Then, the mixture was heated to 90 °C and stirred at this temperature for 7 h. Afterwards, the mixture was cooled down to 5 °C using an ice/water-bath. The mixture was acidified to pH = 2-3 with 1 M aq. HCl. The precipitated solid was filtered off, washed with water and dried under high vacuum. Trimethylated acetyl phloroglucinol **11** (12.9 g, 61.6 mmol, 69%) was isolated as an off-white powder and used without further purification.

^{1}H NMR (600 MHz, DMSO-d_6): δ = 18.96 (s, 1H), 2.46 (s, 3H), 1.77 (s, 3H), 1.27 (s, 6H) ppm.

^{13}C NMR (151 MHz, DMSO-d_6): δ = 199.4, 196.0, 188.9, 176.0, 105.1, 101.9, 48.2, 27.8, 24.3, 7.2 ppm.

The spectroscopic data are in accordance with the literature.[8]

8.1.2 4-Acetyl-6-(3,7-dimethylocta-2,6-dien-1-yl)-5-hydroxy-2,2,6-trimethylcyclohex-4-ene-1,3-dione (13)

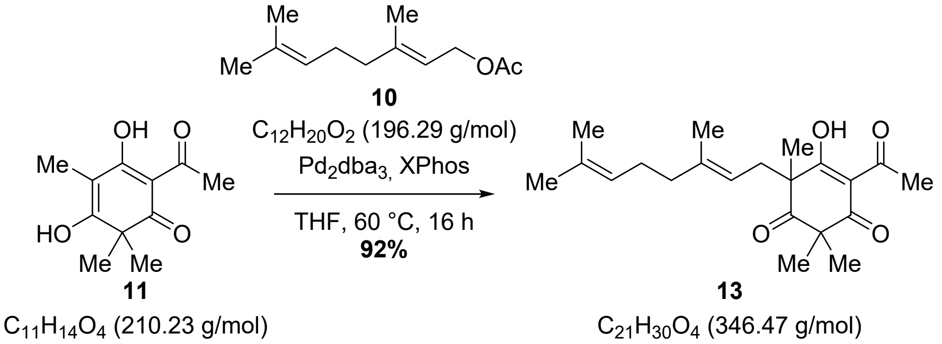

A Schlenk flask was loaded with trimethylated acetyl-phloroglucinol **11** (11.78 g, 56.0 mmol, 1.0 equiv.), $Pd_2(dba)_3$ (1.28 g, 1.40 mmol, 2.5 mol%) and XPhos (2.67 g, 5.60 mmol, 10 mol%). The flask was evacuated and backfilled with argon three times. Afterwards, anhydrous THF (500 mL) and geranyl acetate (**10**, 18.1 mL, 16.5 g, 84.1 mmol, 1.5 equiv.) were added subsequently and the mixture was heated to 60 °C. After stirring at this temperature for 16 h, the mixture was allowed to cool down to RT and filtered through a plug of silica. The silica was thoroughly rinsed with CH_2Cl_2. The filtrate was concentrated under reduced pressure and the crude product was purified by column chromatography (SiO_2, EtOAc:pentane/2% to 5%) to afford geranylated acetyl phloroglucinol **13** (17.8 g, 51.4 mmol, 92%) as a yellowish oil.

The product was isolated as a mixture of two interconverting tautomers (tautomer A : tautomer B = 1:0.7) which gave two sets of signals in the ^{1}H- and ^{13}C-NMR in which many signals overlapped.

Tautomer A:

^{1}H NMR (600 MHz, $CDCl_3$): δ = 18.23 (s, 1H), 4.98 – 4.95 (m, 1H), 4.75 – 4.71 (m, 1H), 2.70 – 2.66 (m, 1H), 2.57 (s, 3H), 2.50 – 2.47 (m, 1H), 1.95 – 1.82 (m, 4H), 1.62 (s, 3H), 1.53 (s, 3H), 1.48 (s, 3H), 1.43 (s, 3H), 1.28 (s, 3H), 1.26 (s, 3H) ppm.

^{13}C NMR (151 MHz, $CDCl_3$): δ = 210.3, 201.8, 197.9, 196.6, 140.8, 131.7, 123.9, 117.3, 111.3, 57.1, 56.2, 39.9, 38.7, 27.8, 26.5, 26.2, 25.7, 22.6, 20.6, 17.7, 16.2 ppm.

Tautomer B

^{1}H NMR (600 MHz, $CDCl_3$): δ = 18.19 (s, 1H), 4.98 – 4.95 (m, 1H), 4.79 – 4.76 (m, 1H), 2.59 – 2.55 (m, 1H), 2.57 (s, 3H), 2.42 – 2.39 (m, 1H), 1.95 – 1.82 (m, 4H), 1.62 (s, 3H), 1.53 (s, 3H), 1.51 (s, 3H), 1.40 (s, 3H), 1.38 (s, 3H), 1.30 (s, 3H) ppm.

^{13}**C NMR** (151 MHz, $CDCl_3$): δ = 210.0, 201.5, 199.3, 196.5, 139.6, 131.7, 123.9, 118.0, 110.7, 60.9, 52.2, 39.9, 38.2, 27.5, 26.6, 26.4, 25.7, 22.0, 21.2, 17.7, 16.3 ppm.

HRMS (ESI, pos.): *m/z* calcd for $C_{21}H_{31}O_4^+$ $[M+H]^+$: 347.2217, found 347.2216.

IR (ATR): $\tilde{\nu}$ = 2979, 2927, 2874, 1717, 1667, 1555, 1426, 1378, 1360, 1326, 1288, 1234, 1178, 1128, 1108, 1027, 955, 932, 863, 834, 804, 781, 741 cm^{-1}.

8.1.3 4-Cinnamoyl-6-(3,7-dimethylocta-2,6-dien-1-yl)-5-hydroxy-2,2,6-trimethylcyclohex-4-ene-1,3-dione (6)

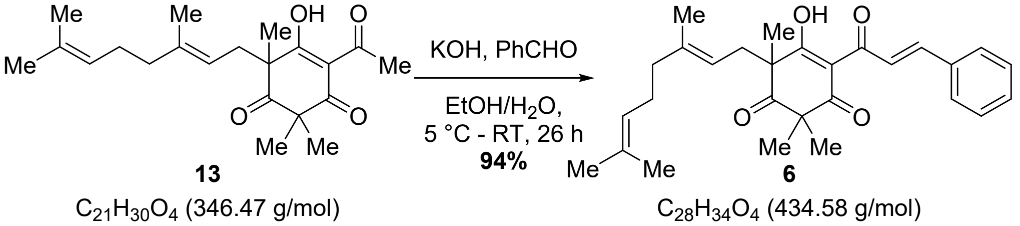

In a round-bottom flask, geranylated acetyl phloroglucinol **13** (17.8 g, 51.4 mmol, 1.0 equiv.) and freshly distilled benzaldehyde (20.8 mL, 21.8 g, 206 mmol, 4.0 equiv.) were dissolved in EtOH (175 mL). The mixture was cooled down to 5 °C using an ice/water-bath and a solution of KOH (7 M, 176 mL) mixed with EtOH (75 mL) was added dropwise through a dropping funnel. After complete addition, the mixture was allowed to warm up to RT and stirred for 18 h. Afterwards, more benzaldehyde (10.4 mL, 10.9 g, 103 mmol, 2.0 equiv.) was added and stirring was continued. After 8 h, crude ^{1}H-NMR indicated complete consumption of the starting material. The mixture was cooled down to 5 °C using an ice/water-bath and a sat. aq. solution of NH_4Cl (500 mL) was added dropwise through the dropping funnel. The mixture was extracted with cyclohexane (3 x 300 mL). The combined organic layers were dried over Na_2SO_4, filtered and concentrated under reduced pressure. The residue was dried while stirring under high vacuum for 3 h to remove parts of the excess benzaldehyde. The crude product was purified by column chromatography (SiO_2, EtOAc:pentane/1:9). After purification, remaining traces of benzaldehyde were removed by stirring under high vacuum at 40 °C over night to afford cinnamoyl phloroglucinol **6** (20.9 g, 48.2 mmol, 94%) as an orange, viscous oil.

The product was isolated as a mixture of two interconverting tautomers (tautomer A : tautomer B = 1:0.8) which gave two sets of signals in the ^{1}H- and ^{13}C-NMR.

Tautomer A:

^{1}**H NMR** (600 MHz, $CDCl_3$): δ = 18.38 (s, 1H), 8.05 – 7.97 (m, 2H), 7.67 – 7.65 (m, 2H), 7.42 – 7.39 (m, 3H), 5.00 – 4.95 (m, 1H), 4.83 – 4.79 (m, 1H), 2.70 – 2.67 (m, 1H), 2.53 – 2.49 (m,

1H), 1.96 – 1.90 (m, 2H), 1.88 – 1.84 (m, 2H), 1.62 (s, 3H), 1.53 (s, 3H), 1.51 (s, 3H), 1.46 (s, 3H), 1.35 (s, 3H), 1.34 (s, 3H) ppm.

^{13}C NMR (151 MHz, $CDCl_3$): δ = 210.2, 201.8, 197.3, 186.3, 146.7, 140.8, 134.9, 131.7, 131.3, 129.2, 129.1, 124.0, 121.5, 117.3, 110.1, 57.9, 57.8, 39.9, 39.0, 26.6, 26.5, 25.7, 21.9, 20.7, 17.7, 16.3 ppm.

Tautomer B:

^{1}H NMR (600 MHz, $CDCl_3$): δ = 18.11 (s, 1H), 8.05 – 7.97 (m, 2H), 7.67 – 7.65 (m, 2H), 7.42 – 7.39 (m, 3H), 5.00 – 4.95 (m, 1H), 4.88 – 4.85 (m, 1H), 2.65 – 2.61 (m, 1H), 2.46 – 2.42 (m, 1H), 1.96 – 1.90 (m, 2H), 1.88 – 1.84 (m, 2H), 1.63 (s, 3H), 1.51 (s, 6H), 1.44 (s, 3H), 1.40 (s, 3H), 1.38 (s, 3H) ppm.

^{13}C NMR (151 MHz, $CDCl_3$): δ = 209.8, 202.7, 197.4, 185.7, 146.7, 139.8, 134.9, 131.7, 131.3, 129.2, 129.1, 124.0, 121.2, 117.8, 109.8, 61.4, 54.1, 39.9, 38.5, 26.6, 26.2, 25.7, 21.3, 21.0, 17.7, 16.4 ppm.

HRMS (ESI, pos.): *m/z* calcd for $C_{28}H_{34}NaO_4^+$ $[M+Na]^+$: 457.2349, found 457.2344.

IR (ATR): $\tilde{\nu}$ = 3101, 3058, 2979, 2920, 2878, 2857, 1717, 1665, 1620, 1577, 1518, 1448, 1412, 1378, 1326, 1268, 1209, 1180, 1157, 1108, 1069, 1027, 979, 959, 945, 873, 851, 836, 793, 752 cm^{-1}.

8.1.4 2-(6-Chloro-3,7-dimethylocta-2,7-dien-1-yl)-4-cinnamoyl-5-hydroxy-2,6,6-trimethylcyclohex-4-ene-1,3-dione (14)

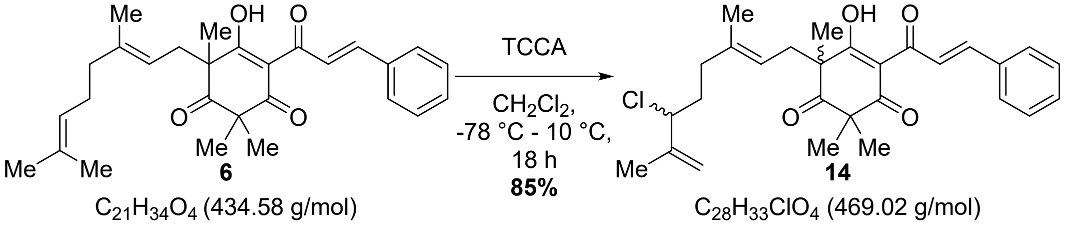

Gram-scale:

In a Schlenk flask geranylated cinnamoyl phloroglucinol **6** (20.9 g, 48.1 mmol, 1.0 equiv.) was dissolved in anhydrous CH_2Cl_2 (500 mL) and the mixture was cooled down to -78 °C using a dry ice/isopropanol bath. Then, trichloroisocyanuric acid (TCCA, 5.15 g, 22.2 mmol, 0.46 equiv.) was added and the mixture was allowed to warm up to 10 °C over 18 h. Afterwards, the mixture was diluted with pentane (500 mL) and filtered through a plug of silica. The silica was thoroughly rinsed with EtOAc:pentane/1:9. The filtrate was concentrated under reduced

pressure and the crude product was purified by column chromatography (SiO_2, Et_2O:pentane/1:19) to afford allylic chloride **14** (11.2 g, 23.9 mmol, 50%) as a yellow oil.

Milligram-scale:

According to the above described procedure geranylated cinnamoyl phloroglucinol **6** (98.0 mg, 226 µmol, 1.0 equiv.) was reacted with trichloroisocyanuric acid (24.1 mg, 104 µmol, 0.46 equiv.) in CH_2Cl_2 (2.3 mL). The mixture was diluted with pentane and filtered through a plug of silica which was rinsed with EtOAc:pentane/1:9 until all of the yellow colour was washed out of the silica to afford the pure allylic chloride **14** (90.0 mg, 192 µmol, 85%) as a yellow oil.

The product was isolated as a mixture of two diastereomers which were also interconverting tautomers. Therefore, there are four sets of signals in the ^{1}H- and ^{13}C-NMR. As a result of this, many signals are overlapping which makes a distinguishment between the different sets very complicated. The signals are given without assignment.

^{1}H NMR (600 MHz, $CDCl_3$): δ = 18.34 – 18.11 (m, 1H), 8.04 – 7.99 (m, 2H), 7.67 – 7.65 (m, 2H), 7.43 – 7.41 (m, 3H), 4.96 – 4.93 (m, 1H), 4.92 – 4.83 (m, 1H), 4.83 – 4.79 (m, 1H), 4.25 – 4.20 (m, 1H), 2.71 – 2.60 (m, 1H), 2.54 – 2.42 (m, 1H), 2.01 – 1.95 (m, 1H), 1.91 – 1.85 (m, 1H), 1.84 – 1.75 (m, 2H), 1.74 – 1.71 (m, 3H), 1.52 (s, 3H), 1.47 – 1.44 (m, 3H), 1.41 – 1.38 (m, 3H), 1.36 – 1.34 (m, 3H) ppm.

^{13}C NMR (176 MHz, $CDCl_3$): δ = 210.1, 210.1, 209.7, 209.7, 202.7, 201.8, 201.8, 197.3, 197.2, 197.2, 197.1, 186.3, 186.2, 185.8, 185.8, 146.9, 146.9, 146.9, 144.3, 144.3, 144.3, 139.3, 139.3, 138.2, 134.8, 134.8, 131.4, 131.3, 129.3, 129.2, 129.2, 129.2, 129.2, 129.2, 129.1, 129.1, 121.4, 121.4, 121.1, 121.1, 119.1, 119.0, 118.6, 118.6, 114.4, 114.3, 114.3, 110.1, 109.6, 109.6, 66.2, 66.2, 66.1, 57.9, 57.8, 57.8, 54.1, 54.1, 38.7, 38.7, 38.0, 38.0, 37.0, 36.9, 36.9, 34.7, 34.7, 34.6, 26.5, 26.5, 26.3, 26.3, 22.2, 22.1, 21.5, 21.5, 21.2, 21.2, 20.7, 17.1, 17.0, 16.3, 16.2 ppm.

HRMS (ESI, pos.): *m/z* calcd for $C_{28}H_{33}ClNaO_4^+$ $[M+Na]^+$: 491.1959, found 491.1966.

IR (ATR): $\tilde{\nu}$ = 3101, 3058, 2979, 2920, 2878, 2857, 1717, 1665, 1620, 1577, 1518, 1448, 1412, 1378, 1326, 1268, 1209, 1180, 1157, 1108, 1069, 1027, 979, 959, 945, 873, 851, 836, 793, 752 cm^{-1}.

8.1.5 4-Cinnamoyl-2-(3,7-dimethylocta-2,5,7-trien-1-yl)-5-hydroxy-2,6,6-trimethylcyclohex-4-ene-1,3-dione (9)

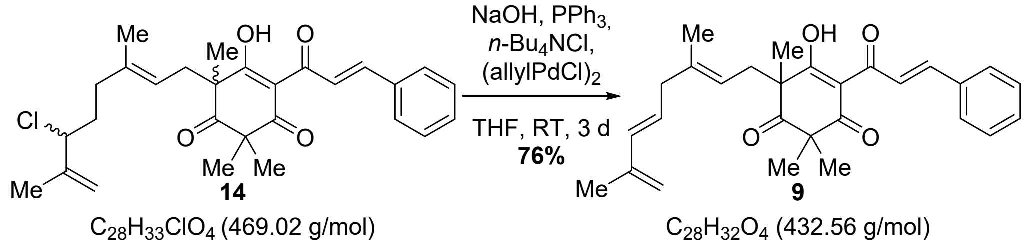

A Schlenk flask was loaded with freshly ground NaOH (2.34 g, 58.6 mmol, 2.5 equiv.), PPh_3 (308 mg, 1.17 mmol, 5 mol%) and allylpalladium chloride dimer (85.8 mg, 235 µmol, 1 mol%). The Schlenk-flask was evacuated and backfilled with argon three times. Afterwards, anhydrous THF (40 mL) and a solution of tetrabutylammonium chloride (40 mM, 16.8 mL, 704 µmol, 3 mol%) in anhydrous THF were added subsequently. The mixture was stirred vigorously and a solution of allylic chloride **14** (11.0 g, 23.5 mmol, 1.0 equiv.) in anhydrous THF (50 mL) was added slowly while keeping the mixture at 20 °C using a water bath. Then, the mixture was stirred at RT for 3 d. Afterwards, water (100 mL) was added and the mixture was extracted with EtOAc (3 x 100 mL). The combined organic layers were washed with water, brine, dried over Na_2SO_4, filtered and concentrated under reduced pressure. The crude product was purified by column chromatography (SiO_2, EtOAc:cyclohexane/1:9) to afford diene **9** (7.74 g, 17.8 mmol, 76%) as a yellow oil.

The product was isolated as a mixture of two interconverting tautomers (tautomer A : tautomer B = 1:0.75) which gave two sets of signals in the ^{1}H- and ^{13}C-NMR.

Tautomer A:

^{1}H NMR (600 MHz, $CDCl_3$): δ = 18.42 (s, 1H), 8.04 – 7.98 (m, 2H), 7.67 – 7.65 (m, 2H), 7.43 – 7.40 (m, 3H), 6.05 – 6.00 (m, 1H), 5.46 – 5.40 (m, 1H), 4.90 – 4.80 (m, 3H), 2.73 – 2.66 (m, 1H), 2.64 – 2.61 (m, 2H), 2.54 – 2.44 (m, 1H), 1.75 (s, 3H), 1.51 (s, 3H), 1.48 (s, 3H), 1.35 (s, 3H), 1.33 (s, 3H) ppm.

^{13}C NMR (151 MHz, $CDCl_3$): δ = 210.3, 201.7, 197.2, 186.4, 146.8, 142.0, 139.7, 134.9, 134.8, 131.3, 129.2, 129.1, 127.7, 121.5, 118.3, 115.0, 110.3, 58.0, 57.6, 43.0, 39.2, 26.4, 22.3, 20.3, 18.7, 16.5 ppm.

Tautomer B:

^{1}H NMR (600 MHz, $CDCl_3$): δ = 18.12 (s, 1H), 8.04 – 7.98 (m, 2H), 7.67 – 7.65 (m, 2H), 7.43 – 7.40 (m, 3H), 6.05 – 6.00 (m, 1H), 5.46 – 5.40 (m, 1H), 4.90 – 4.80 (m, 3H), 2.73 – 2.66 (m, 1H), 2.64 – 2.61 (m, 2H), 2.54 – 2.44 (m, 1H), 1.74 (s, 3H), 1.51 (s, 3H), 1.42 (s, 3H), 1.40 (s, 6H) ppm.

^{13}C NMR (151 MHz, $CDCl_3$): δ = 210.0, 202.7, 197.4, 185.7, 146.8, 141.9, 138.6, 134.9, 134.8, 131.3, 129.2, 129.2, 127.6, 121.2, 118.9, 115.1, 109.9, 61.1, 54.3, 43.0, 38.6, 26.6, 21.6, 21.1, 18.7, 16.6 ppm.

HRMS (ESI, pos.): *m/z* calcd for $C_{28}H_{32}NaO_4^+$ $[M+Na]^+$: 455.2193, found 455.2201.

IR (ATR): $\tilde{\nu}$ = 3101, 3082, 3024, 2980, 2931, 2871, 2853, 1717, 1666, 1620, 1577, 1519, 1449, 1415, 1380, 1327, 1305, 1266, 1209, 1181, 1158, 1118, 1026, 966, 946, 884, 854, 839, 794, 752 cm^{-1}.

8.2 Convergent Route to Intermediate 9

8.2.1 6-Chloro-3,7-dimethylocta-2,7-dien-1-yl acetate (S-1)

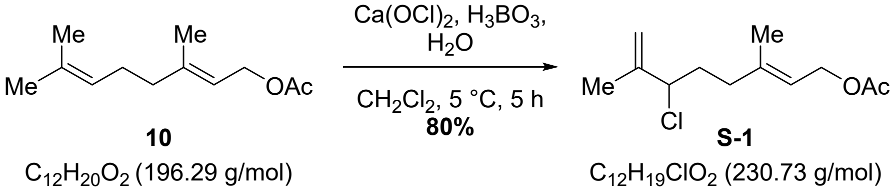

Calcium hypochlorite (67 w%, 6.47 g, 30.6 mmol, 0.6 equiv.) and boric acid (7.58 g, 122 mmol, 2.4 equiv.) were suspended in CH_2Cl_2 (50 mL) in an Erlenmeyer flask. The resulting mixture was cooled down to 5 °C using an ice-water bath and geranyl acetate (**10**) (11.0 mL, 10.0 g, 50.9 mmol, 1.0 equiv.) was added. Under vigorous stirring, water (13.8 mL) was added over 5 h. After complete conversion of the starting material, solid Na_2SO_3 was added, the inorganic salts were filtered off through a plug of celite and the filtrate was concentrated under reduced pressure. The crude product was purified by column chromatography (SiO_2, EtOAc/pentane, 1:19 to 1:9) to afford allylic chloride **S-1** (9.37 g, 40.6 mmol, 80%) as a colorless oil.

^{1}H NMR (600 MHz, $CDCl_3$): δ = 5.37 (tq, *J* = 7.0, 1.3 Hz, 1H), 5.01 – 5.00 (m, 1H), 4.90 (p, *J* = 1.5 Hz, 1H), 4.58 (d, *J* = 7.1 Hz, 2H), 4.35 – 4.32 (m, 1H), 2.19 – 2.13 (m, 2H), 2.05 (s, 3H), 2.01 – 1.88 (m, 2H), 1.81 (s, 3H), 1.71 (s, 3H) ppm.

^{13}**C NMR** (151 MHz, $CDCl_3$): δ = 171.2, 144.3, 140.7, 119.5, 114.5, 66.3, 61.3, 36.6, 34.5, 21.2, 17.1, 16.6 ppm.

The spectroscopic data are in accordance with the literature.[9]

8.2.2 3,7-Dimethylocta-2,5,7-trien-1-yl acetate (S-2)

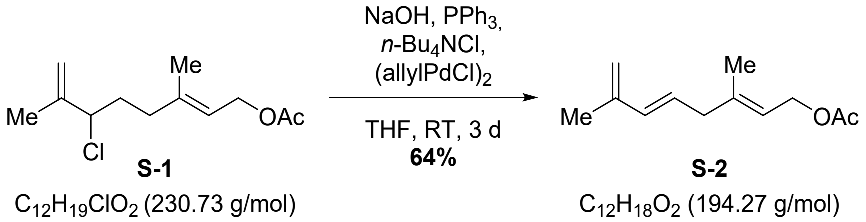

The dehydrochlorination of allylic chloride **S-1** was performed according to a literature known procedure.[10] Freshly ground NaOH (3.57 g, 89.2 mmol, 1.1 equiv.), PPh_3 (1.06 g, 4.05 mmol, 5 mol%) and *n*-Bu_4NCl (676 mg, 2.43 mmol, 3 mol%) were loaded into a Schlenk flask. The flask was evacuated and backfilled with argon three times. Afterwards, anhydrous THF (60 mL) and allylic chloride **S-1** (18.7 g, 81.0 mmol, 1.0 equiv.) dissolved in anhydrous THF (100 mL) were added to the mixture subsequently while keeping the temperature under 25 °C. The mixture was stirred for three days. Afterwards, water (100 mL) was added, the phases were separated and the aqueous phase was extracted with diethyl ether (3 x 100 mL). The combined organic phases were washed with water, brine, dried over Na_2SO_4, filtered and concentrated under reduced pressure. The crude product was purified by column chromatography (SiO_2, EtOAc/pentane, 1:19) to afford diene **S-2** (10.0 g, 51.5 mmol, 64%) as a yellowish oil.

^{1}**H NMR** (600 MHz, $CDCl_3$): δ = 6.16 (d, J = 15.6 Hz, 1H), 5.61 (dt, J = 15.5, 7.1 Hz, 1H), 5.37 (tq, J = 7.1, 1.4 Hz, 1H), 4.91 – 4.90 (m, 2H), 4.60 (d, J = 7.3 Hz, 2H), 2.82 (d, J = 7.1 Hz, 2H), 2.06 (s, 3H), 1.84 (s, 3H), 1.70 (s, 3H) ppm.

^{13}**C NMR** (151 MHz, $CDCl_3$): δ = 171.3, 142.0, 141.2, 135.0, 127.5, 119.3, 115.3, 61.5, 42.8, 21.2, 18.8, 16.8 ppm.

HRMS (ESI, pos.): *m/z* calcd for $C_{12}H_{18}O_2Na^+$ $[M+Na]^+$: 217.1199, found 217.1193.

IR (ATR): $\tilde{\nu}$ = 3081, 3022, 2975, 2942, 2919, 1737, 1672, 1649, 1608, 1438, 1380, 1365, 1319, 1227, 1109, 1021, 966, 884, 826, 793, 777, 747, 719 cm^{-1}.

8.2.3 4-Acetyl-6-(3,7-dimethylocta-2,5,7-trien-1-yl)-5-hydroxy-2,2,6-trimethylcyclohex-4-ene-1,3-dione (S-3)

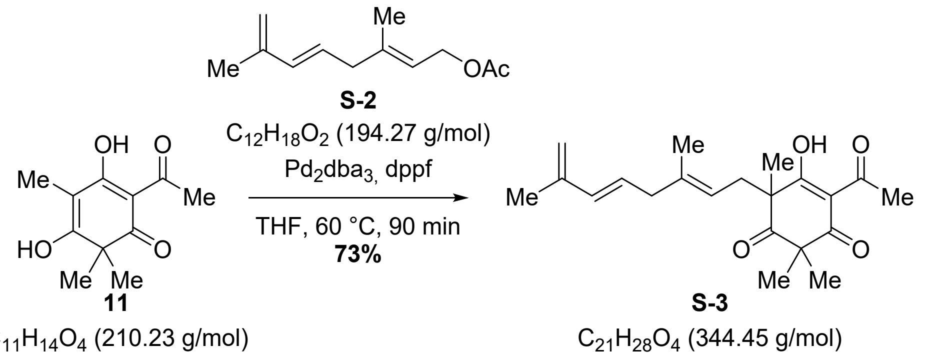

A Schlenk flask was loaded with trimethylated acetyl-phloroglucinol **11** (5.80 g, 27.6 mmol, 1.0 equiv.), $Pd_2(dba)_3$ (632 mg, 690 µmol, 2.5 mol%) and dppf (765 mg, 1.38 mmol, 5 mol%). The flask was evacuated and backfilled with argon three times. Afterwards, anhydrous THF (200 mL) and allylic acetate **S-2** (8.04 g, 41.4 mmol, 1.5 equiv.) were added subsequently and the mixture was heated to 60 °C. After stirring at this temperature for 90 min, the mixture was allowed to cool down to RT and filtered through a plug of silica. The silica was thoroughly rinsed with EtOAc. The filtrate was concentrated under reduced pressure and the crude product was purified by column chromatography (SiO_2, Et_2O/pentane, 1:19 to 1:9) to afford diene **S-3** (6.90 g, 20.0 mmol, 73%) as a yellowish oil.

The product was isolated as a mixture of two interconverting tautomers (tautomer A : tautomer B = 1:0.7) which gave two sets of signals in the ^{1}H- and ^{13}C-NMR.

Tautomer A:

^{1}H NMR (600 MHz, CD_2Cl_2): δ = 18.23 (s, 1H), 6.08 – 6.04 (m, 1H), 5.51 – 5.43 (m, 1H), 4.88 – 4.78 (m, 3H), 2.73 – 2.58 (m, 3H), 2.56 (s, 3H), 2.53 – 2.41 (m, 1H), 1.80 (s, 3H), 1.50 (s, 3H), 1.45 (s, 3H), 1.29 (s, 3H), 1.24 (s, 3H) ppm.

^{13}C NMR (151 MHz, CD_2Cl_2): δ = 210.5, 202.2, 197.9, 196.6, 142.5, 139.8, 134.9, 128.1, 118.7, 115.0, 111.7, 57.6, 56.3, 43.2, 39.0, 27.8, 26.4, 23.0, 20.4, 18.8, 16.5 ppm.

Tautomer B

^{1}H NMR (600 MHz, CD_2Cl_2): δ = 18.20 (s, 1H), 6.08 – 6.04 (m, 1H), 5.51 – 5.43 (m, 1H), 4.88 – 4.78 (m, 3H), 2.73 – 2.58 (m, 3H), 2.56 (s, 3H), 2.53 – 2.41 (m, 1H), 1.79 (s, 3H), 1.53 (s, 3H), 1.39 (s, 6H), 1.31 (s, 3H) ppm.

^{13}**C NMR** (151 MHz, CD_2Cl_2): δ = 210.2, 201.7, 199.6, 196.5, 142.4, 138.6, 134.9, 128.2, 119.4, 115.1, 111.1, 60.9, 52.5, 43.2, 38.4, 27.6, 26.6, 22.0, 21.6, 18.8, 16.5 ppm.

HRMS (ESI, pos.): *m/z* calcd for $C_{21}H_{28}NaO_4^+$ $[M+Na]^+$: 367.1880, found 367.1896.

IR (ATR): $\tilde{\nu}$ = 3082, 2976, 2936, 2874, 2853, 1739, 1718, 1670, 1607, 1556, 1453, 1434, 1381, 1364, 1323, 1229, 1177, 1124, 1025, 966, 928, 882, 861, 836, 794, 779, 743, 722 cm^{-1}.

8.2.4 4-Cinnamoyl-6-(3,7-dimethylocta-2,5,7-trien-1-yl)-5-hydroxy-2,2,6-trimethylcyclohex-4-ene-1,3-dione (9)

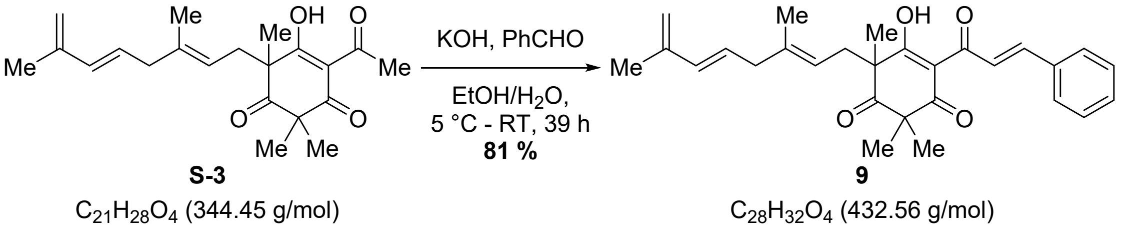

In a round-bottom flask, a mixture of aq. KOH (7 M, 15 mL, 24 equiv.) and EtOH (15 mL) was cooled down to 5 °C using an ice-water bath. Acetyl phloroglucinol **S-3** (1.70 g, 4.94 mmol, 1.0 equiv.) and benzaldehyde (1.00 mL, 1.05 g, 9.87 mmol, 2.0 equiv.) dissolved in EtOH (15 mL) were added slowly. Then, the mixture was allowed to warm up to RT. After stirring the mixture at this temperature for 16 h, more benzaldehyde (1.00 mL, 1.05 g, 9.87 mmol, 2 equiv.) was added. Stirring was continued for 7 h. Then, more benzaldehyde (1.00 mL, 1.05 g, 9.87 mmol, 2 equiv.) was added. After another 16 h the starting material was consumed completely. The mixture was poured into an ice-cold sat. aq. solution of ammonium chloride (100 mL) and extracted with EtOAc (3 x 100 mL). The combined organic layers were washed with water, brine, dried over Na_2SO_4, filtered and concentrated under reduced pressure. The residue was dried while stirring under high vacuum for 3 h to remove parts of the excess benzaldehyde. The crude product was purified by column chromatography (SiO_2, Et_2O/pentane, 1:19). After purification, the product was once more dried while stirring under high vacuum over night to remove the remaining traces of benzaldehyde to afford cinnamoyl phloroglucinol **9** (1,73 g, 4.00 mmol, 81%) as a yellow, thick oil.

The product was isolated as a mixture of two interconverting tautomers (tautomer A : tautomer B = 1:0.75) which gave two sets of signals in the ^{1}H- and ^{13}C-NMR.

Tautomer A:

^{1}**H NMR** (600 MHz, $CDCl_3$): δ = 18.42 (s, 1H), 8.04 – 7.98 (m, 2H), 7.67 – 7.65 (m, 2H), 7.43 – 7.40 (m, 3H), 6.05 – 6.00 (m, 1H), 5.46 – 5.40 (m, 1H), 4.90 – 4.80 (m, 3H), 2.73 – 2.66 (m, 1H), 2.64 – 2.61 (m, 2H), 2.54 – 2.44 (m, 1H), 1.75 (s, 3H), 1.51 (s, 3H), 1.48 (s, 3H), 1.35 (s, 3H), 1.33 (s, 3H) ppm.

^{13}**C NMR** (151 MHz, $CDCl_3$): δ = 210.3, 201.7, 197.2, 186.4, 146.8, 142.0, 139.7, 134.9, 134.8, 131.3, 129.2, 129.1, 127.7, 121.5, 118.3, 115.0, 110.3, 58.0, 57.6, 43.0, 39.2, 26.4, 22.3, 20.3, 18.7, 16.5 ppm.

Tautomer B:

^{1}**H NMR** (600 MHz, $CDCl_3$): δ = 18.12 (s, 1H), 8.04 – 7.98 (m, 2H), 7.67 – 7.65 (m, 2H), 7.43 – 7.40 (m, 3H), 6.05 – 6.00 (m, 1H), 5.46 – 5.40 (m, 1H), 4.90 – 4.80 (m, 3H), 2.73 – 2.66 (m, 1H), 2.64 – 2.61 (m, 2H), 2.54 – 2.44 (m, 1H), 1.74 (s, 3H), 1.51 (s, 3H), 1.42 (s, 3H), 1.40 (s, 6H) ppm.

^{13}**C NMR** (151 MHz, $CDCl_3$): δ = 210.0, 202.7, 197.4, 185.7, 146.8, 141.9, 138.6, 134.9, 134.8, 131.3, 129.2, 129.2, 127.6, 121.2, 118.9, 115.1, 109.9, 61.1, 54.3, 43.0, 38.6, 26.6, 21.6, 21.1, 18.7, 16.6 ppm.

HRMS (ESI, pos.): *m/z* calcd for $C_{28}H_{32}NaO_4^+$ $[M+Na]^+$: 455.2193, found 455.2201.

IR (ATR): $\tilde{\nu}$ = 3101, 3082, 3024, 2980, 2931, 2871, 2853, 1717, 1666, 1620, 1577, 1519, 1449, 1415, 1380, 1327, 1305, 1266, 1209, 1181, 1158, 1118, 1026, 966, 946, 884, 854, 839, 794, 752 cm^{-1}.

8.3 IMDA and Backbone Oxidation

8.3.1 IMDA-diastereomers 15 and 8

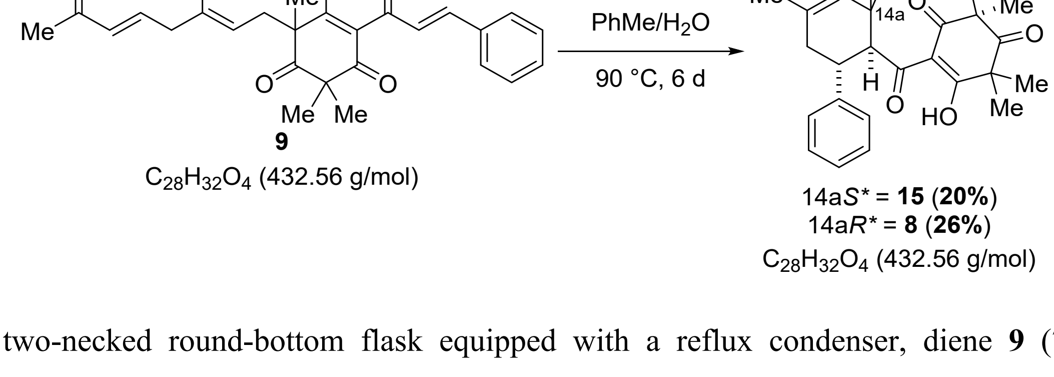

In a two-necked round-bottom flask equipped with a reflux condenser, diene **9** (7.70 g, 17.8 mmol, 1.0 equiv.) was dissolved in toluene (500 mL). Water (500 mL) was added and the mixture was degassed by bubbling through a stream of argon while stirring vigorously for 5 h.

Afterwards, the mixture was heated to 90 °C for 6 d. After allowing the mixture to cool down to RT, the phases were separated and the aqueous phase was extracted with EtOAc (3 x 100 mL). The combined organic layers were washed with brine, dried over Na_2SO_4, filtered and concentrated under reduced pressure. Pentane was added to the residue and the mixture was filtered. The solids were collected, cyclohexane was added and the suspension was sonicated for 30 min. The mixture was filtered again and the solid was dried in the high vacuum to afford diastereomer **8** (2.00 g, 4.64 mmol, 26%) as a white, amorphous solid. The filtrate of the second filtration was concentrated under reduced pressure, pentane was added and the suspension was sonicated for 10 min. The solid was filtered off and dried in the high vacuum to afford diastereomer **15** (1.55 g, 3.58 mmol, 20%) as a white, amorphous solid.

Analytical data for compound **15**:

^{1}H NMR (600 MHz, $CDCl_3$): δ = 17.50 (s, 1H), 7.40 – 7.39 (m, 2H), 7.19 – 7.16 (m, 2H), 7.08 – 7.05 (m, 1H), 5.20 – 5.18 (m, 1H), 5.00 – 4.97 (m, 1H), 3.44 (dd, *J* = 11.0, 9.8 Hz, 1H), 3.19 (td, *J* = 11.7, 4.4 Hz, 1H), 3.12 – 3.06 (m, 1H), 2.41 – 2.34 (m, 1H), 2.33 – 2.29 (m, 1H), 2.19 (dd, *J* = 13.8, 12.1 Hz, 1H), 2.14 – 2.10 (m, 1H), 1.99 – 1.95 (m, 1H), 1.80 (t, *J* = 12.5 Hz, 1H), 1.72 (s, 3H), 1.40 (s, 3H), 1.34 (s, 3H), 1.33 (s, 3H), 0.98 (s, 3H) ppm.

^{13}C NMR (151 MHz, $CDCl_3$): δ = 206.3, 202.6, 196.4, 195.8, 142.3, 140.5, 133.6, 128.9, 127.9, 126.4, 124.8, 120.1, 114.4, 60.5, 52.0, 49.0, 48.3, 46.0, 42.2, 38.6, 37.1, 28.2, 23.4, 20.0, 16.6, 15.5 ppm.

HRMS (ESI, pos.): *m/z* calcd for $C_{28}H_{32}KO_4^+$ $[M+K]^+$: 471.1933, found 471.1914.

IR (ATR): $\tilde{\nu}$ = 3029, 2979, 2913, 2878, 2850, 1722, 1684, 1539, 1495, 1454, 1387, 1376, 1361, 1345, 1310, 1217, 1162, 1141, 1103, 1082, 1025, 950, 923, 907, 897, 881, 840, 818, 800, 753 cm^{-1}.

X-ray: Crystals were grown by the vapor diffusion technique of a solution of **15** in CH_2Cl_2 with *n*-pentane at ambient temperature.

Analytical data for compound **8**:

^{1}H NMR (600 MHz, $CDCl_3$): δ = 17.96 (s, 1H), 7.37 – 7.32 (m, 4H), 7.23 – 7.21 (m, 1H), 5.18 (brs, 1H), 4.59 – 4.56 (m, 1H), 3.55 – 3.54 (m, 1H), 3.38 – 3.36 (m, 1H), 2.86 – 2.83 (m, 1H), 2.81 – 2.76 (m, 1H), 2.38 (t, *J* = 12.7 Hz, 1H), 2.29 – 2.26 (m, 1H), 2.24 – 2.18 (m, 2H), 1.89 (s, 3H), 1.78 – 1.74 (m, 1H), 1.45 (s, 3H), 1.44 (s, 3H), 1.38 (s, 3H), 1.16 (s, 3H) ppm.

^{13}C NMR (151 MHz, $CDCl_3$): δ = 209.2, 201.3, 200.7, 194.7, 144.9, 134.8, 134.4, 128.5, 127.3, 126.5, 124.9, 123.4, 117.0, 59.1, 56.0, 43.4, 43.0, 39.5, 39.5, 32.1, 30.8, 28.2, 23.6, 22.9, 16.7, 14.2 ppm.

HRMS (ESI, pos.): *m/z* calcd for $C_{28}H_{32}NaO_4^+$ $[M+Na]^+$: 455.2193, found 455.2181.

IR (ATR): $\tilde{\nu}$ = 3062, 3024, 2979, 2906, 2852, 1722, 1675, 1541, 1495, 1466, 1450, 1370, 1360, 1316, 1296, 1268, 1216, 1180, 1153, 1081, 1068, 1030, 993, 958, 930, 881, 841, 809, 756, 703 cm^{-1}.

X-ray: Crystals were grown by the vapor diffusion technique of a solution of **8** in CH_2Cl_2 with *n*-pentane at ambient temperature.

8.3.2 Epoxide 16

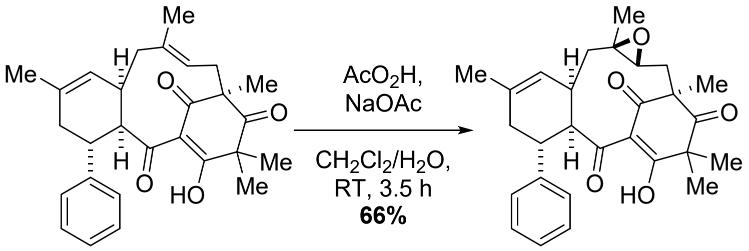

8
$C_{28}H_{32}O_4$ (432.56 g/mol)

16
$C_{28}H_{32}O_5$ (448.56 g/mol)

In a round-bottom flask, **8** (2.00 g, 4.64 mmol, 1.0 equiv.) was dissolved in CH_2Cl_2 (14 mL). 1 M aq. NaOAc (23 mL) was added to the mixture. Under vigorous stirring, acetic acid peroxide (35 w% in acetic acid, 2.95 mL, 3.33 g, 15.3 mmol, 3.33 equiv.) was added over 3.5 h. Afterwards, the mixture was cooled down to 5 °C using an ice/water-bath and sat. aq. $Na_2S_2O_3$ (50 mL) was added slowly under vigorously stirring. Then, the phases were separated and the aqueous phase was extracted with CH_2Cl_2 (3 x 20 mL). The combined organic layers were washed with aq. sat. $NaHCO_3$, water , brine, dried over Na_2SO_4, filtered and concentrated under reduced pressure. The crude product was purified by column chromatography (SiO_2, CH_2Cl_2) to afford epoxide **16** (1.38 g, 3.08 mmol, 66%) as a white, amorphous solid.

^{1}H NMR (600 MHz, CD_2Cl_2): δ = 17.95 (s, 1H), 7.40 – 7.39 (m, 2H), 7.36 – 7.34 (m, 2H), 7.25 – 7.23 (m, 1H), 5.14 (s, 1H), 3.95 (d, *J* = 6.0 Hz, 1H), 3.45 (d, *J* = 6.6 Hz, 1H), 2.80 – 2.75 (m, 1H), 2.68 (d, *J* = 13.2 Hz, 1H), 2.30 – 2.27 (m, 1H), 2.21 – 2.19 (m, 1H), 2.14 – 2.10 (m, 1H), 1.97 (dd, *J* = 13.8, 5.1 Hz, 1H), 1.86 (s, 3H), 1.48 (s, 3H), 1.44 (s, 3H), 1.38 (s, 3H),1.35 – 1.34 (m, 1H), 0.85 (t, *J* = 13.8 Hz, 1H), 0.74 (s, 3H) ppm.

^{13}**C NMR** (151 MHz, CD_2Cl_2): δ = 209.0, 200.0, 199.5, 199.1, 144.9, 134.7, 128.8, 127.5, 126.9, 123.4, 115.5, 64.3, 60.0, 58.6, 57.0, 42.7, 40.7, 39.7, 37.8, 33.2, 30.9, 28.1, 23.5, 23.2, 16.7, 16.1 ppm.

HRMS (ESI, neg.): *m/z* calcd for $C_{28}H_{31}O_5^-$ [M-H]$^-$: 447.2177, found 447.2169.

IR (ATR): $\tilde{\nu}$ = 3024, 2976, 2908, 2861, 1724, 1672, 1542, 1495, 1450, 1427, 1386, 1370, 1318, 1297, 1280, 1254, 1218, 1183, 1166, 1117, 1065, 1050, 1028, 990, 978, 967, 945, 926, 883, 865, 813, 753, 702 cm^{-1}.

8.3.3 (±)-Cleistocaltone A (1)

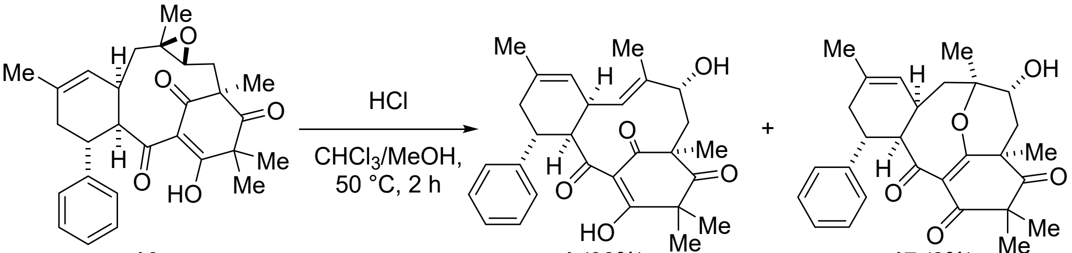

16 $C_{28}H_{32}O_5$ (448.56 g/mol) — **1** (**33%**) $C_{28}H_{32}O_5$ (448.56 g/mol) — **17** (**9%**) $C_{28}H_{32}O_5$ (448.56 g/mol)

In a two-necked round-bottom flask equipped with a dropping funnel, epoxide **16** (1.74 g, 4.02 mmol, 1.0 equiv.) was dissolved in $CHCl_3$ (150 mL). The flask was capped and the mixture was heated to 50 °C. HCl (1 w% in MeOH, 92.9 mL, 20.1 mmol, 5.0 equiv.) was added through the dropping funnel and the mixture was stirred for 2 h. Afterwards, the mixture was concentrated under reduced pressure. The crude product was purified by flash column chromatography (SiO_2, EtOAc/pentane, 1:4) to afford (±)-Cleistocaltone A (**1**) (587 mg, 1.30 mmol, 33%) as a white amorphous solid and side product **17** (162 mg, 361 μmol, 9%) as a white amorphous solid.

Analytical data of (±)-Cleistocaltone A (**1**):

^{1}**H NMR** (700 MHz, $CDCl_3$): δ = 16.46 (s, 1H), 7.33 – 7.31 (m, 2H), 7.24 – 7.22 (m, 1H), 7.21 – 7.20 (m, 2H), 5.09 – 5.08 (m, 1H), 4.85 (dd, *J* = 9.8, 1.6 Hz, 1H), 4.22 – 4.21 (m, 1H), 3.75 (dd, *J* = 11.4, 2.8 Hz, 1H), 3.48 – 3.46 (m, 1H), 3.06 – 3.02 (m, 2H), 2.50 (dd, *J* = 13.1, 2.8 Hz, 1H), 2.21 – 2.18 (m, 1H), 2.13 (dd, *J* = 13.2, 11.4 Hz, 1H), 1.81 (s, 3H), 1.50 (s, 3H), 1.47 (s, 3H), 1.32 (s, 3H), 1.18 (d, *J* = 1.4 Hz, 3H) ppm.

^{13}**C NMR** (151 MHz, $CDCl_3$): δ = 210.9, 207.1, 196.4, 187.3, 146.0, 138.2, 136.2, 131.5, 128.6, 127.4, 126.5, 120.3, 116.3, 75.0, 56.7, 51.0, 47.9, 45.2, 38.1, 32.3, 32.0, 28.8, 27.5, 23.7, 20.1, 8.9 ppm.

HRMS (ESI, pos.): *m/z* calcd for $C_{28}H_{32}NaO_5^+$ $[M+Na]^+$: 471.2142, found 471.2147.

IR (ATR): $\tilde{\nu}$ = 3474, 3394, 2924, 2851, 1716, 1659, 1568, 1495, 1451, 1402, 1378, 1243, 1102, 1031, 956, 864, 767, 737 cm^{-1}.

The spectroscopic data are in accordance with the literature.[11]

Analytical data of sideproduct **17**:

^{1}H NMR (700 MHz, $CDCl_3$) δ = 7.30 – 7.28 (m, 2H), 7.22 – 7.20 (m, 1H), 7.16 – 7.15 (m, 2H), 5.04 (brs, 1H), 3.85 (brs, 1H), 3.59 (dd, *J* = 4.3, 2.2 Hz, 1H), 3.54 (dd, *J* = 5.4, 3.7 Hz, 1H), 2.72 – 2.68 (m, 1H), 2.46 – 2.44 (m, 1H), 2.34 (dd, *J* = 15.0, 2.2 Hz, 1H), 2.15 – 2.09 (m, 3H), 2.05 – 2.01 (m, 1H), 1.71 (s, 3H), 1.70 (s, 3H), 1.45 (s, 3H), 1.38 (dd, *J* = 15.1, 6.1 Hz, 1H), 1.27 (s, 3H), 1.25 (s, 3H) ppm.

^{13}C NMR (176 MHz, $CDCl_3$) δ = 210.6, 198.1, 195.0, 175.6, 145.5, 135.6, 128.5, 127.6, 127.4, 126.3, 123.7, 89.7, 70.5, 59.1, 56.1, 46.6, 39.0, 37.9, 36.7, 31.9, 27.7, 27.2, 23.9, 23.3, 19.2, 19.1 ppm.

HRMS (ESI, pos.): *m/z* calcd for $C_{28}H_{32}NaO_5^+$ $[M+Na]^+$: 471.2142, found 471.2157.

IR (ATR): $\tilde{\nu}$ = 3467, 2982, 2961, 2924, 2855, 1718, 1689, 1645, 1622, 1495, 1453, 1381, 1353, 1320, 1246, 1220, 1178, 1145, 1092, 1050, 1010, 992, 971, 940, 921, 902, 852, 822, 795, 754, 712, 702 cm^{-1}.

X-ray: Crystals were grown by slow evaporation of a solution of **17** in diethyl ether at ambient temperature. The compound crystallized with one diethyl ether molecule in the cell.

9. Comparison of NMR Data

9.1 (±)-Cleistocaltone A (1)

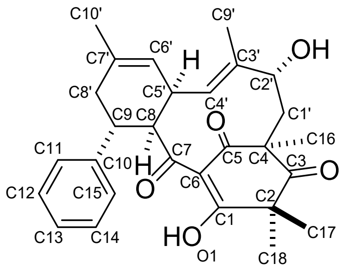

Table S3. Comparison of ^{1}H and ^{13}C NMR Data for Isolated and Synthetic (±)-Cleistocaltone A (1).[a]

No.	Isolation[b] ^{13}C NMR (125 MHz) δ_C/ppm[c]	Synthetic ^{13}C NMR (176 MHz) δ_C/ppm[c]	Δ/ppm	Isolation[b] ^{1}H NMR (500 MHz) δ_H/ppm (*J* in Hz)[d]	Synthetic ^{1}H NMR (700 MHz) δ_H/ppm (*J* in Hz)[c]	Δ/ppm
1	187.1	187.3	0.2			
2	50.9	51.0	0.1			
3	210.9	210.9	0.0			
4	56.6	56.7	0.1			
5	196.2	196.4	0.2			
6	116.2	116.3	0.1			
7	206.9	207.1	0.2			
8	38.0	38.1	0.1	3.53 m	3.47 m	0.06
9	47.9	47.9	0.0	4.27 m	4.21 m	0.06
10	145.9	146.0	0.1			
11	127.2	127.4	0.2	7.28 m	7.20 m	0.08
12	128.5	128.6	0.1	7.37	7.32	0.05
13	126.4	126.5	0.1	7.28	7.23	0.05
14	128.5	128.6	0.1	7.37	7.32	0.05
15	127.3	127.4	0.1	7.28 m	7.20 m	0.08
16	27.4	27.5	0.1	1.37s	1.32 s	0.05
17	28.6	28.8	0.2	1.56 s	1.50 s	0.06
18	20.0	20.1	0.1	1.52 s	1.47 s	0.05
1'a	45.2	45.2	0.0	2.54 dd (13.2, 2.7)	2.50 dd (13.1, 2.8)	0.04
1'b				2.16 dd (13.2, 11.3)	2.13 dd (13.2, 11.4)	0.03
2'	74.8	75.0	0.2	3.80 dd (11.3, 2.7)	3.75 dd (11.4, 2.8)	0.05
3'	138.3	138.2	0.1			
4'	131.2	131.5	0.3	4.90 d (9.9)	4.85 dd (9.8, 1.6)	0.05
5'	31.9	32.0	0.1	3.10	3.04	0.06
6'	120.3	120.3	0.0	5.15 m	5.09 m	0.06
7'	136.0	136.2	0.2			
8'a	32.2	32.3	0.1	3.10	3.04	0.06
8'b				2.26 m	2.20 m	0.06
9'	8.8	8.9	0.1	1.23 s	1.18 d (1.4)	0.05
10'	23.5	23.7	0.2	1.87 s	1.81 s	0.06
1-OH				16.51 s	16.46 s	0.05

a) All data were obtained in $CDCl_3$. Overlapped signals were reported without designating multiplicity. b) Data from reference 6. c) Chemical shifts are reported relative to the corresponding residual non-deuterated solvent signal ($CDCl_3$: δ_H = 7.26 ppm, δ_C = 77.16 ppm). d) Chemical shifts are reported relative to TMS (δ_H = 0.00 ppm).

10. X-ray data

10.1 IMDA-diastereomer 15 (CCDC2352924)

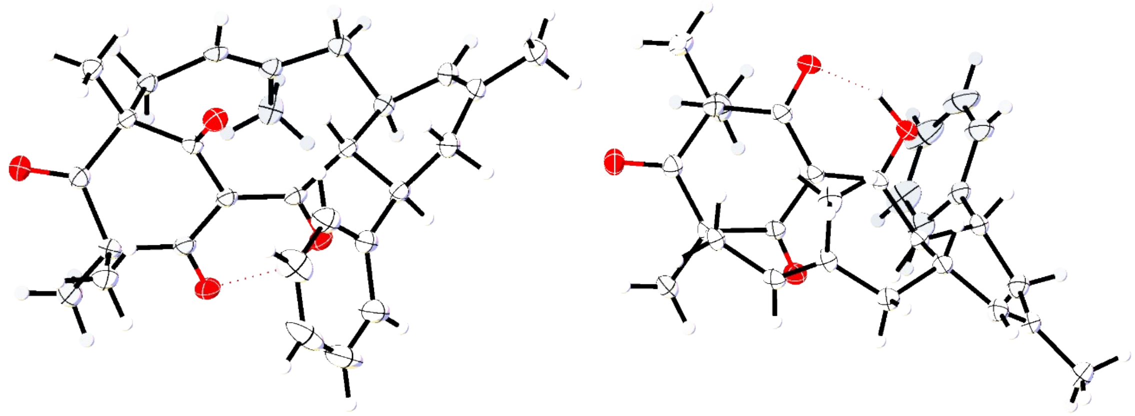

Table S4. Crystal data of IMDA-diastereomer 15

Empirical formula	$C_{28}H_{32}O_4$
Formula weight	432.53
Temperature/K	140(2)
Crystal system	monoclinic
Space group	$P2_1/n$
a/Å	9.53542(6)
b/Å	18.20996(12)
c/Å	14.54563(9)
α/°	90
β/°	105.5556(2)
γ/°	90
Volume/Å³	2433.18(3)
Z	4
ρ_{calc}g/cm³	1.181
μ/mm^{-1}	0.617
F(000)	928.0
Crystal size/mm³	0.44 × 0.25 × 0.14
Radiation	CuKα (λ = 1.54178)
2Θ range for data collection/°	7.96 to 144.238
Index ranges	$-11 \leq h \leq 11$, $-22 \leq k \leq 22$, $-14 \leq l \leq 17$
Reflections collected	33228
Independent reflections	4766 [R_{int} = 0.0376, R_{sigma} = 0.0210]
Data/restraints/parameters	4766/0/296
Goodness-of-fit on F^2	1.049
Final R indexes [I>=2σ (I)]	R_1 = 0.0392, wR_2 = 0.0938
Final R indexes [all data]	R_1 = 0.0402, wR_2 = 0.0943
Largest diff. peak/hole / e Å^{-3}	0.22/-0.30

10.2 IMDA-diastereomer 8 (CCDC2352922)

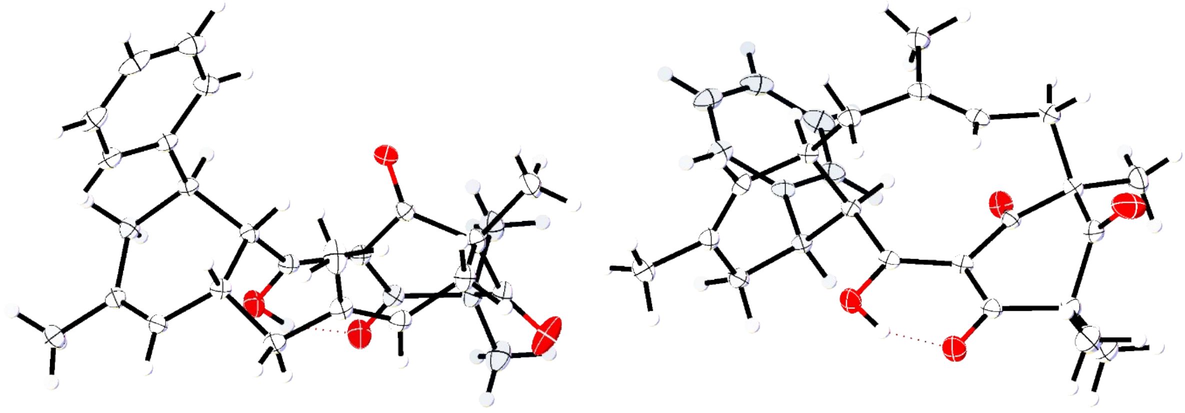

Table S5. Crystal data of IMDA-diastereomer 8

Empirical formula	$C_{28}H_{32}O_4$
Formula weight	432.53
Temperature/K	140(2)
Crystal system	monoclinic
Space group	$P2_1/n$
a/Å	8.38079(5)
b/Å	23.63124(15)
c/Å	12.06328(8)
α/°	90
β/°	106.2413(2)
γ/°	90
Volume/Å^3	2293.77(3)
Z	4
ρ_{calc}g/cm^3	1.253
μ/mm^{-1}	0.655
F(000)	928.0
Crystal size/mm^3	0.39 × 0.23 × 0.1
Radiation	CuKα (λ = 1.54178)
2Θ range for data collection/°	7.482 to 144.09
Index ranges	$-10 \leq h \leq 9$, $-28 \leq k \leq 29$, $-14 \leq l \leq 14$
Reflections collected	34216
Independent reflections	4516 [R_{int} = 0.0478, R_{sigma} = 0.0240]
Data/restraints/parameters	4516/0/296
Goodness-of-fit on F^2	1.046
Final R indexes [I>=2σ (I)]	R_1 = 0.0432, wR_2 = 0.1117
Final R indexes [all data]	R_1 = 0.0439, wR_2 = 0.1123
Largest diff. peak/hole / e Å^{-3}	0.41/-0.31

10.3 Side Product 17 (CCDC2352923)

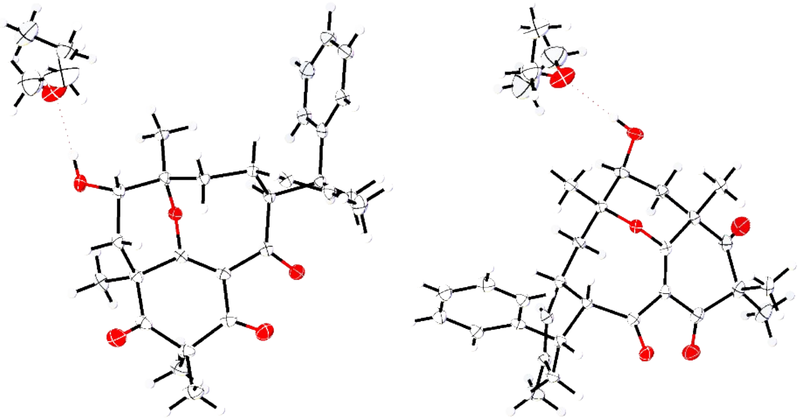

Table S6: Crystal data of side product 17

Empirical formula	$C_{32}H_{42}O_6$
Formula weight	522.65
Temperature/K	150(2)
Crystal system	orthorhombic
Space group	$P2_12_12_1$
a/Å	10.10314(7)
b/Å	10.72852(8)
c/Å	25.70876(18)
α/°	90
β/°	90
γ/°	90
Volume/Å^3	2786.62(3)
Z	4
ρ_{calc}g/cm^3	1.246
μ/mm^{-1}	0.679
F(000)	1128.0
Crystal size/mm^3	0.22 × 0.16 × 0.12
Radiation	CuKα (λ = 1.54178)
2Θ range for data collection/°	6.876 to 140.684
Index ranges	$-12 \leq h \leq 12$, $-13 \leq k \leq 13$, $-31 \leq l \leq 31$
Reflections collected	73519
Independent reflections	5283 [R_{int} = 0.0383, R_{sigma} = 0.0184]
Data/restraints/parameters	5283/0/351
Goodness-of-fit on F^2	1.043
Final R indexes [I>=2σ (I)]	R_1 = 0.0327, wR_2 = 0.0870
Final R indexes [all data]	R_1 = 0.0330, wR_2 = 0.0872
Largest diff. peak/hole / e Å^{-3}	0.60/-0.25
Flack parameter	0.00(3)

11. NMR Spectra

Methylated acetyl-phloroglucinol 11

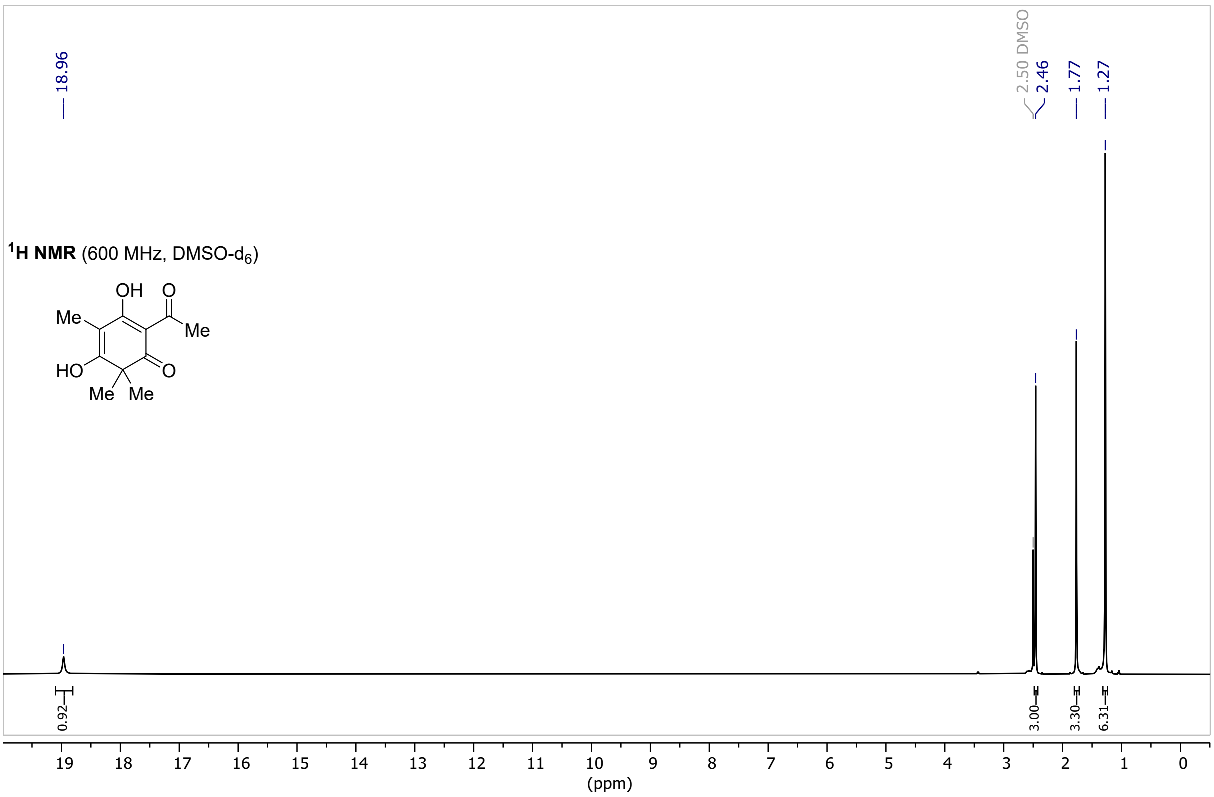

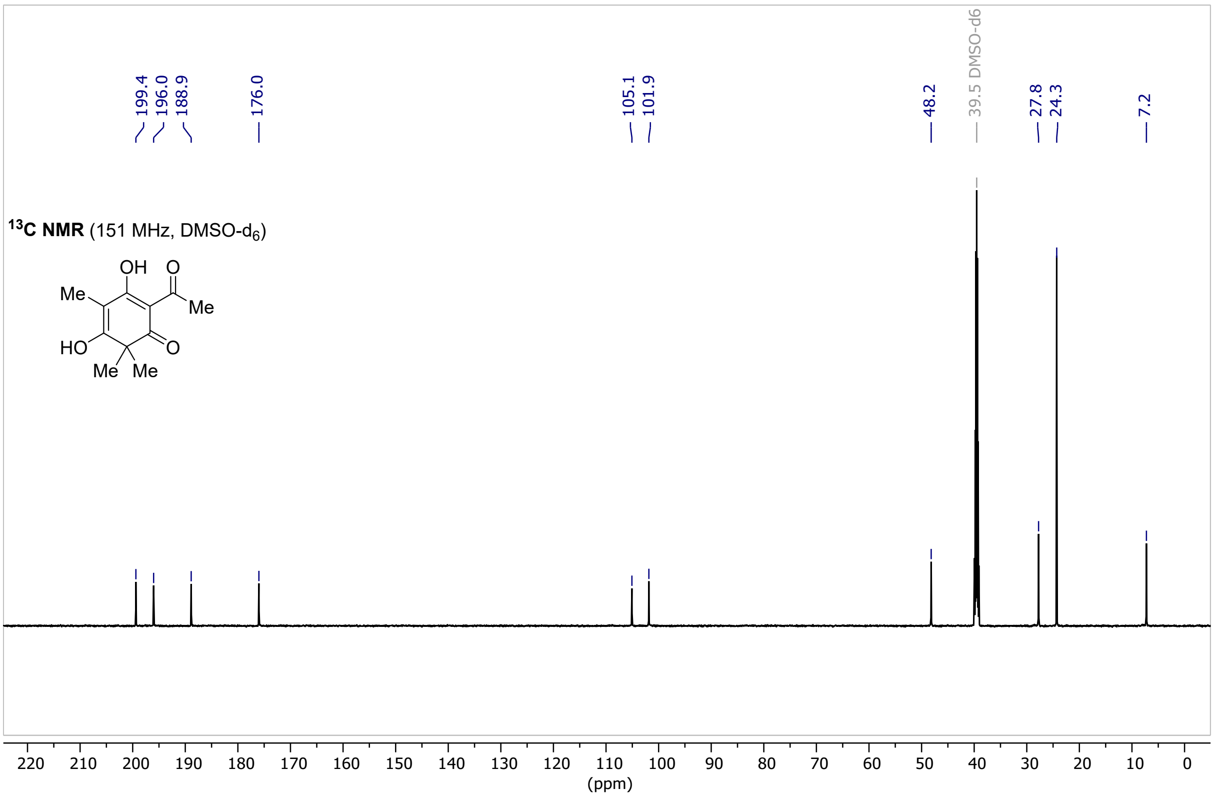

Geranylated acetyl-phloroglucinol 13

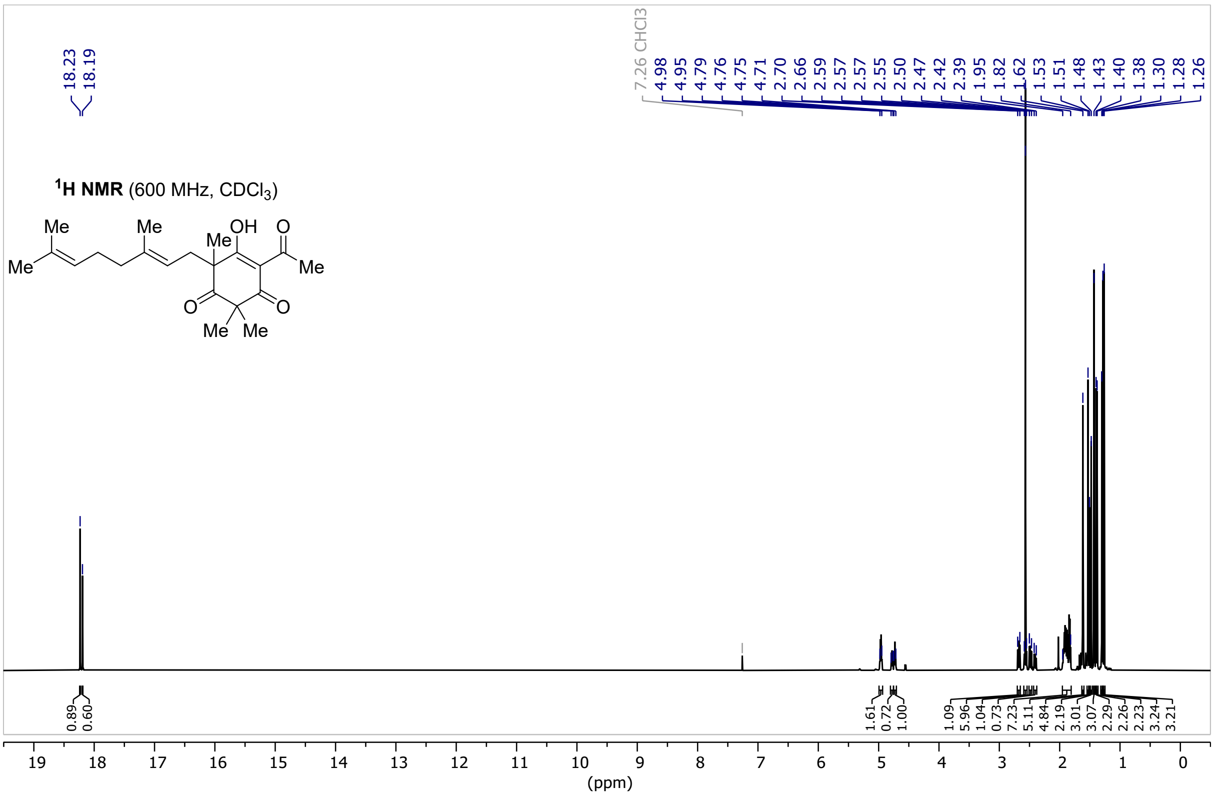

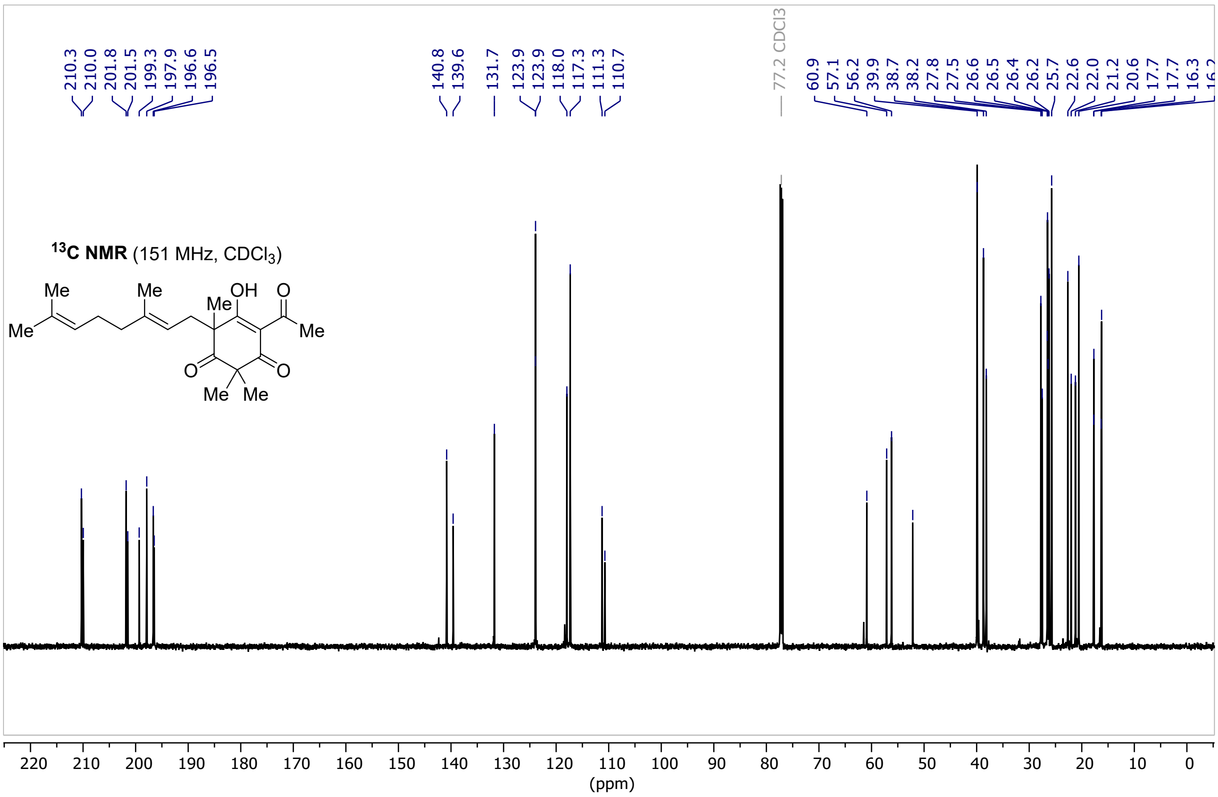

Geranylated cinnamoyl-phloroglucinol 6

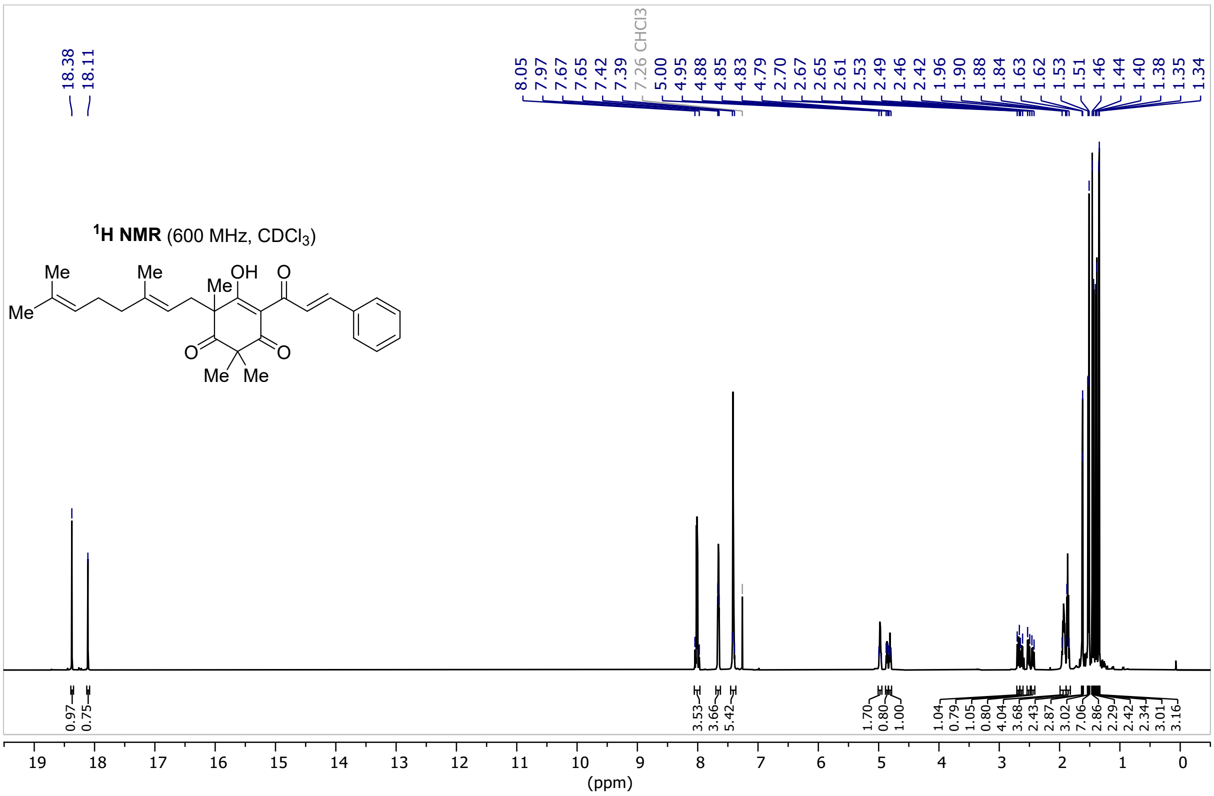

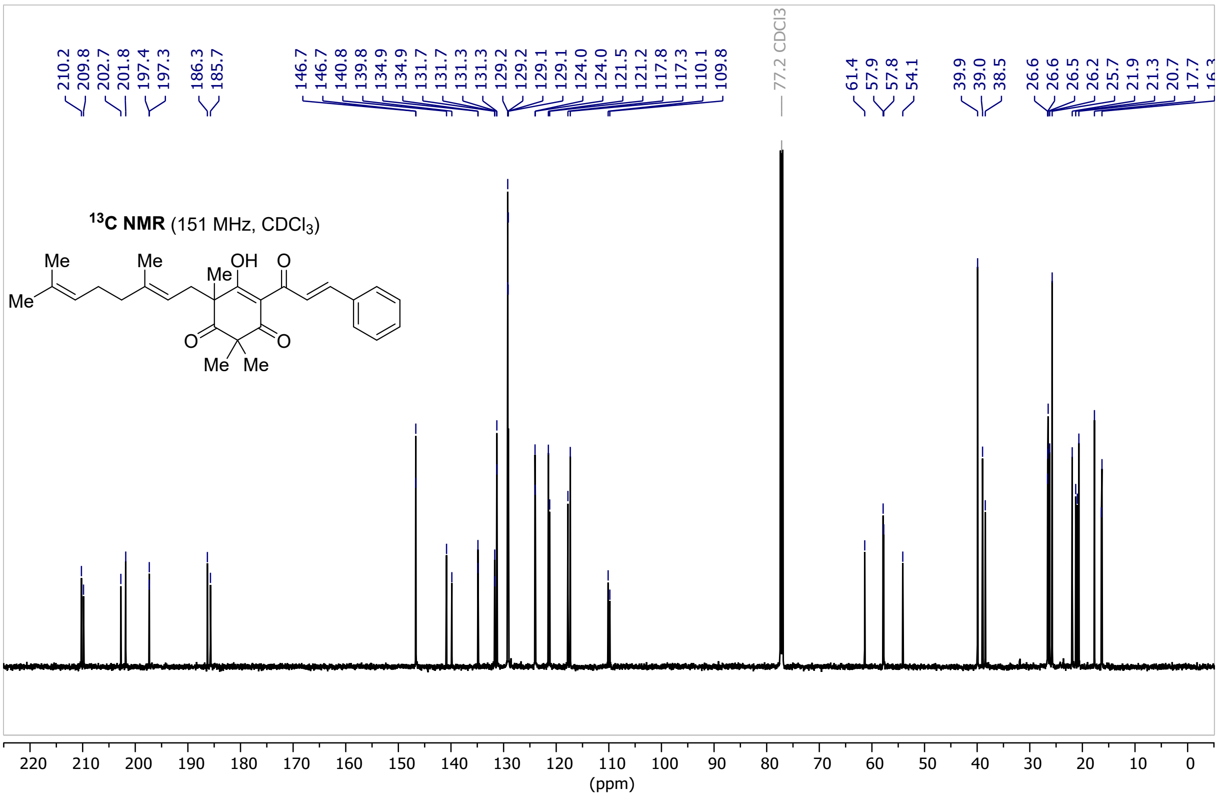

Allylic chloride 14

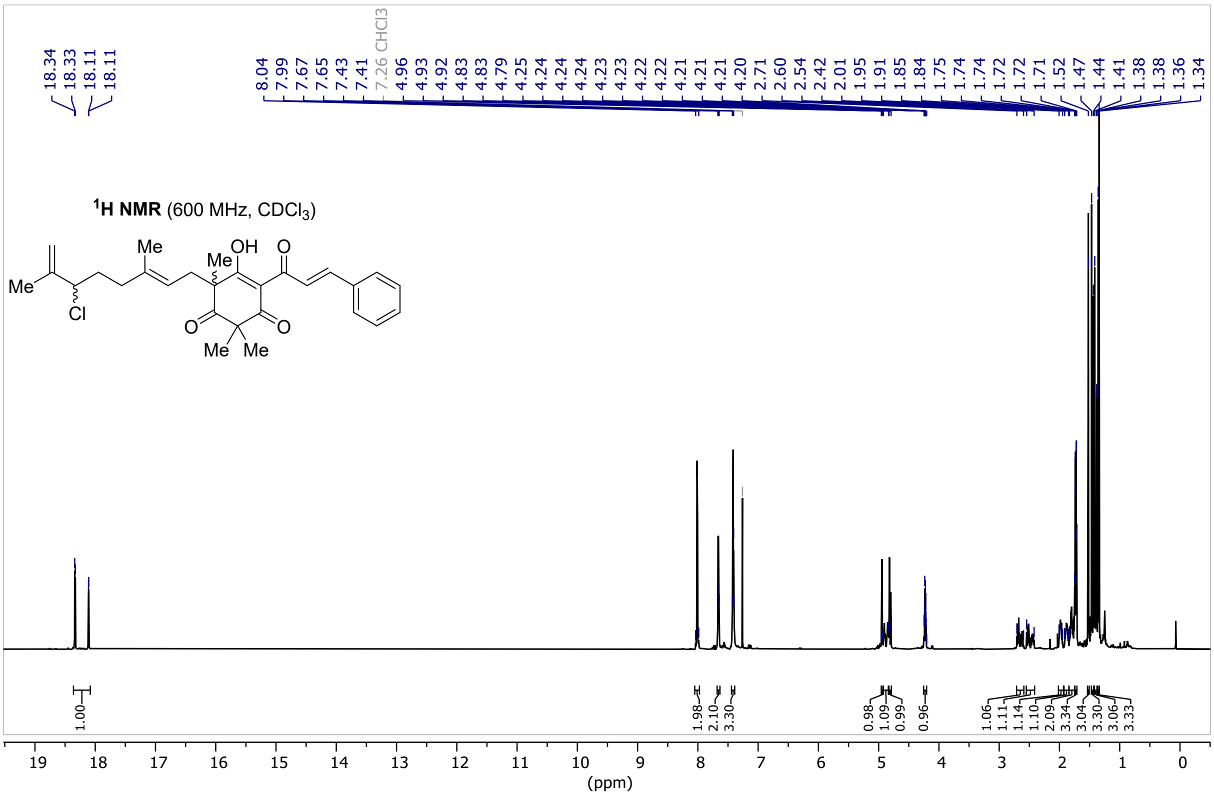

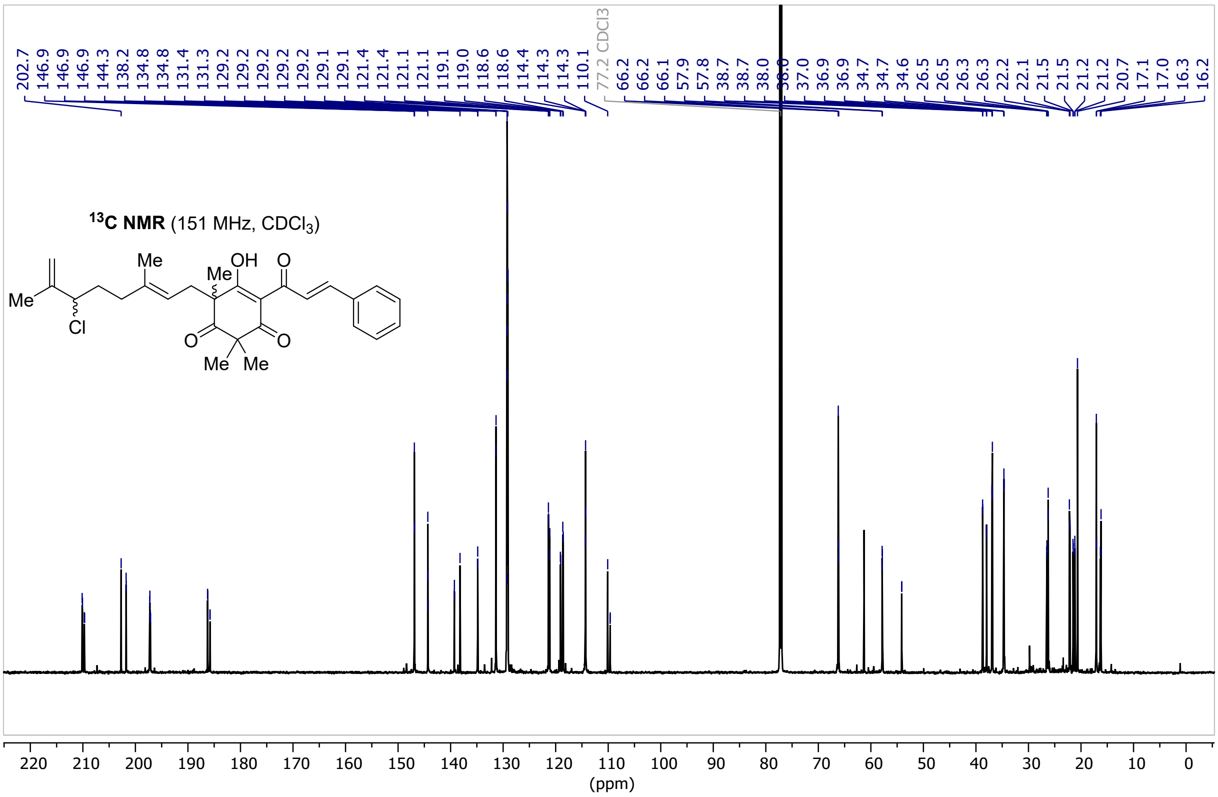

Allylic chloride S-1

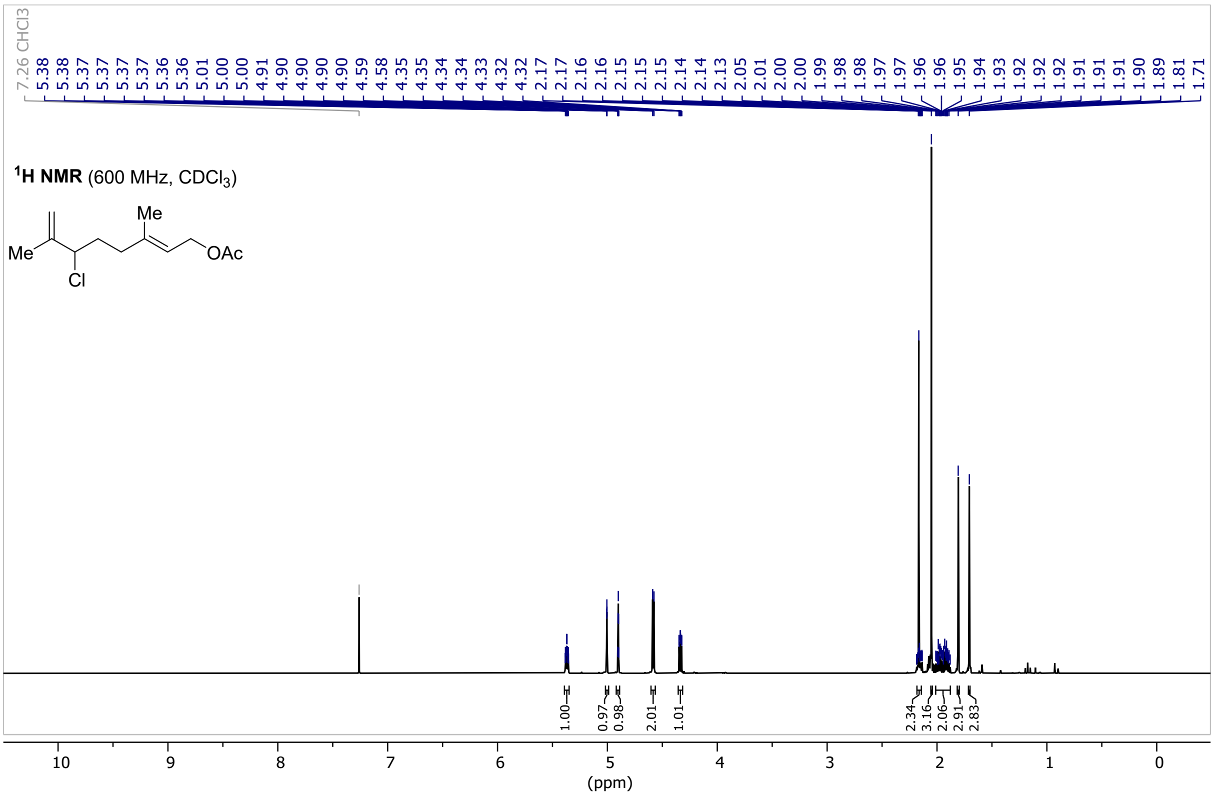

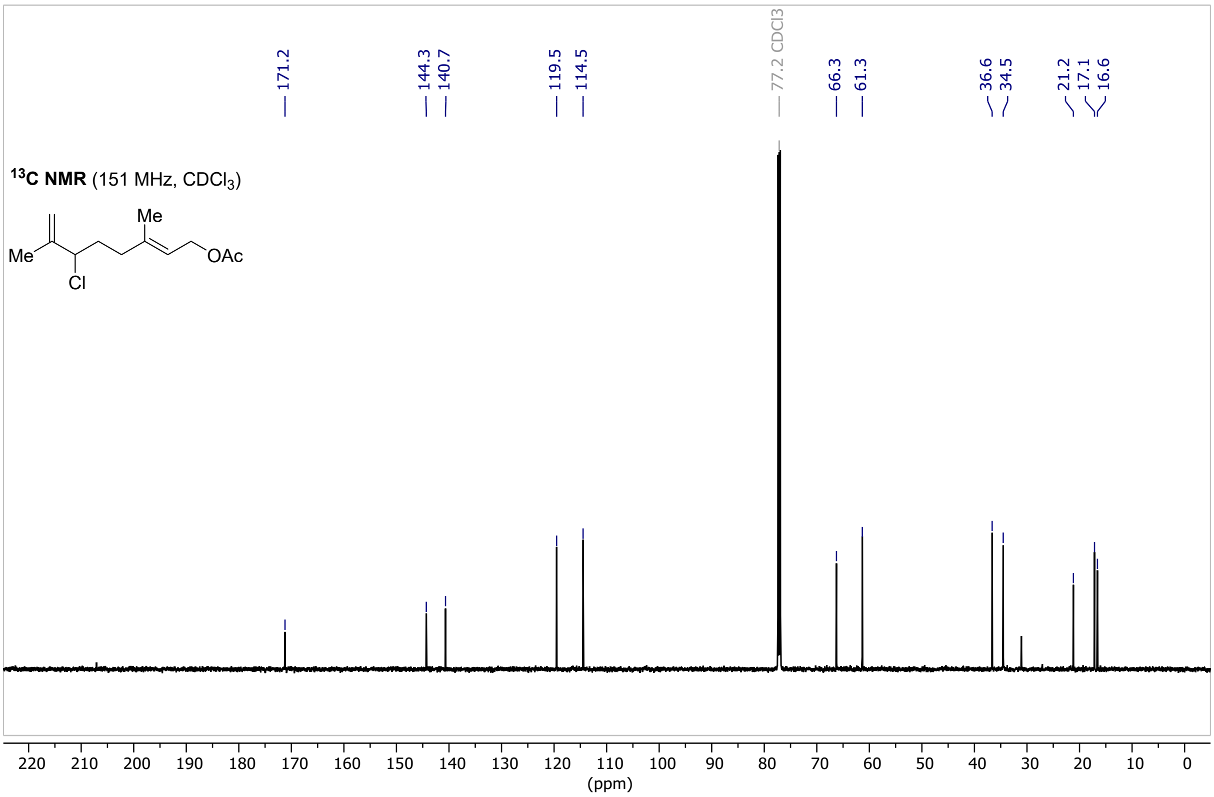

Diene S-2

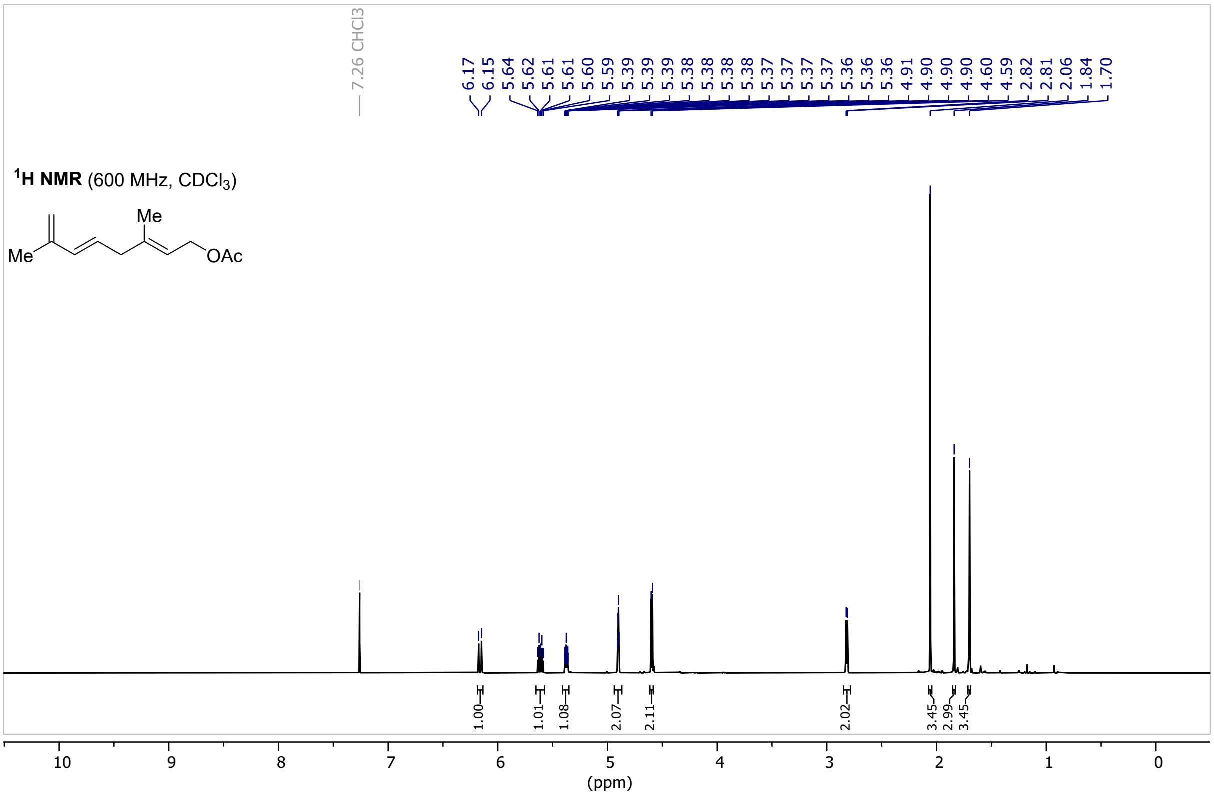

7.26 CHCl3
6.17
6.15
5.64
5.62
5.61
5.61
5.60
5.59
5.39
5.39
5.39
5.38
5.38
5.38
5.38
5.37
5.37
5.37
5.37
5.36
5.36
5.36
4.91
4.90
4.90
4.90
4.60
4.59
2.82
2.81
2.06
1.84
1.70
¹H NMR (600 MHz, CDCl3)
Me
Me
OAc
1.00
1.01
1.08
2.07
2.11
2.02
3.45
2.99
3.45
10
9
8
7
6
5
4
3
2
1
0
(ppm)

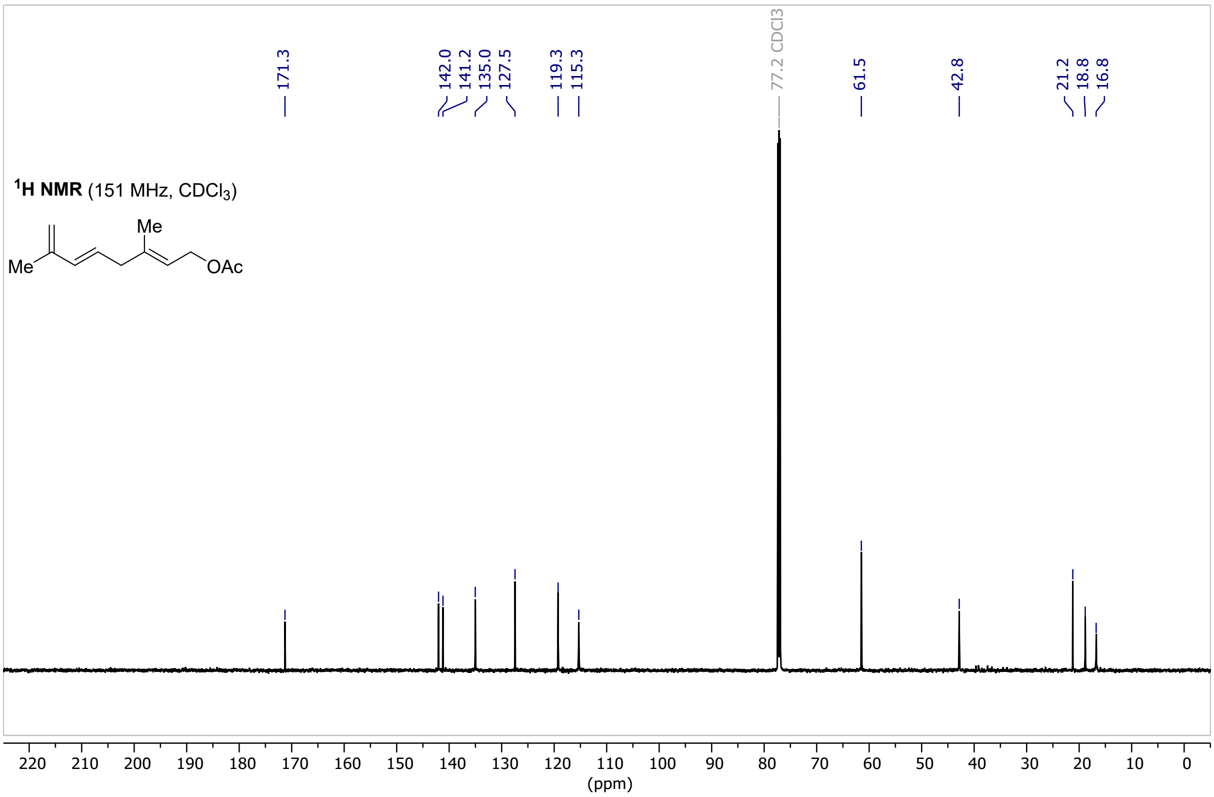

171.3
142.0
141.2
135.0
127.5
119.3
115.3
77.2 CDCl3
61.5
42.8
21.2
18.8
16.8
¹H NMR (151 MHz, CDCl3)
Me
Me
OAc
220
210
200
190
180
170
160
150
140
130
120
110
100
90
80
70
60
50
40
30
20
10
0
(ppm)

Diene S-3

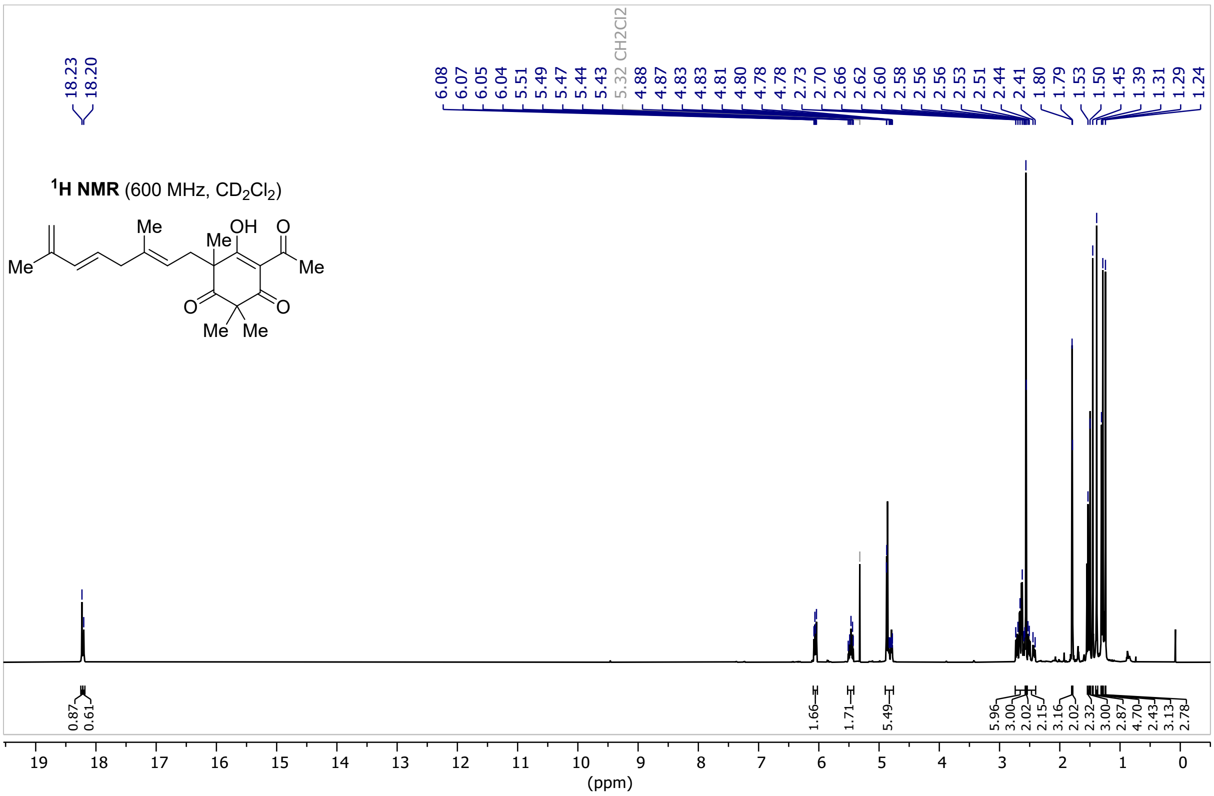

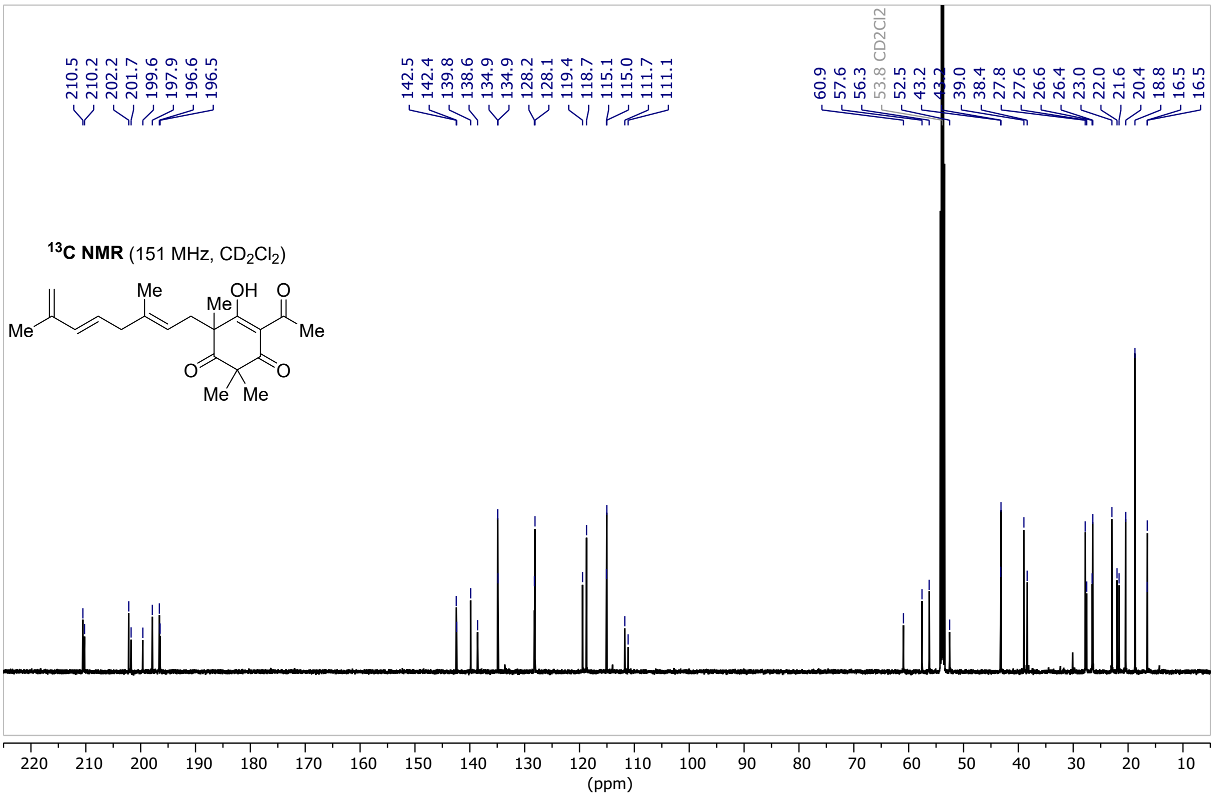

Diene 9

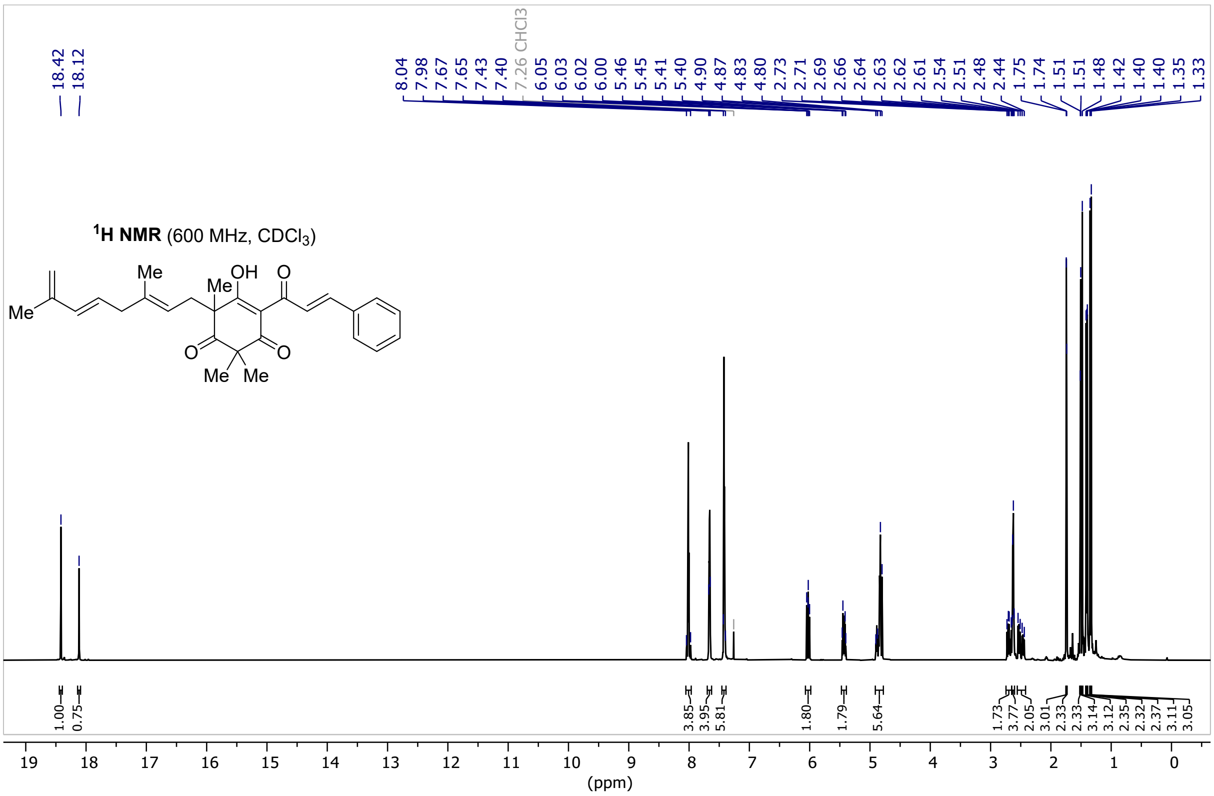

1H NMR (600 MHz, CDCl3)
Me
OH
O
Me
Me
O
O
Me Me
(ppm)

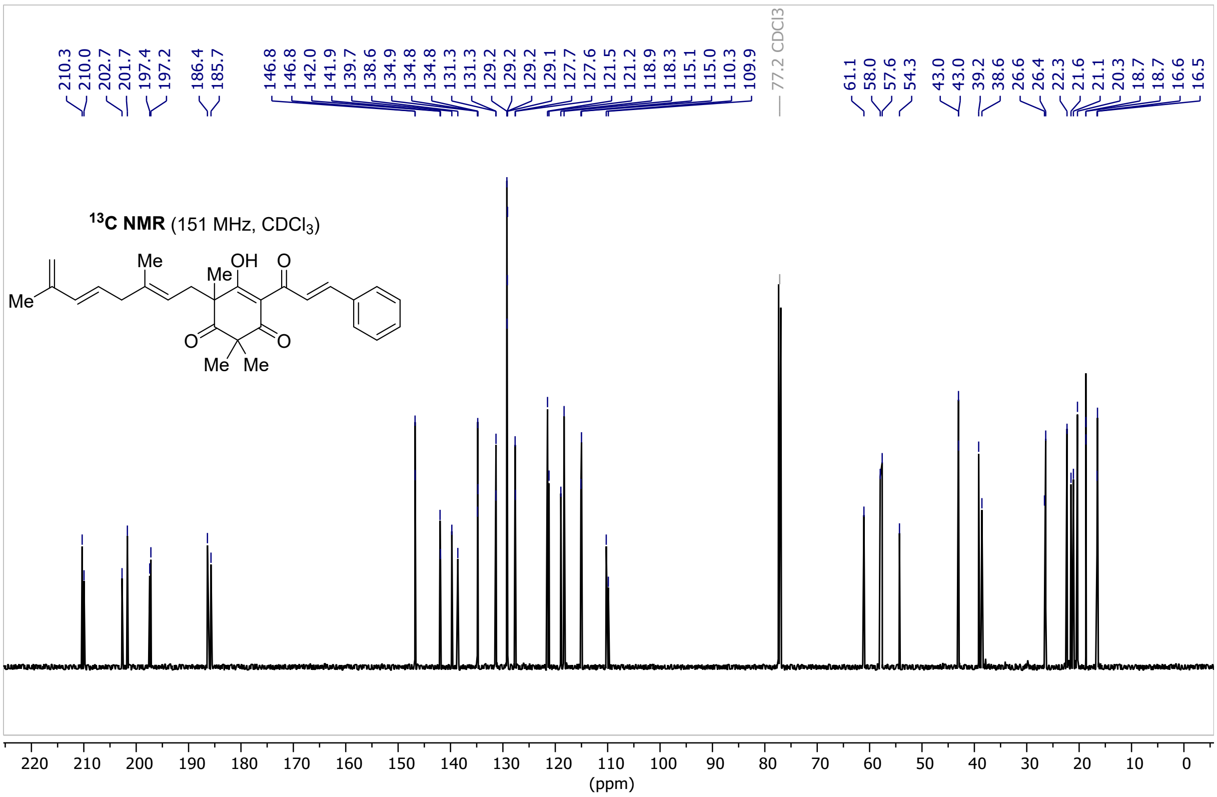

13C NMR (151 MHz, CDCl3)
Me
OH
O
Me
Me
O
O
Me Me
(ppm)

IMDA Diastereomer 15

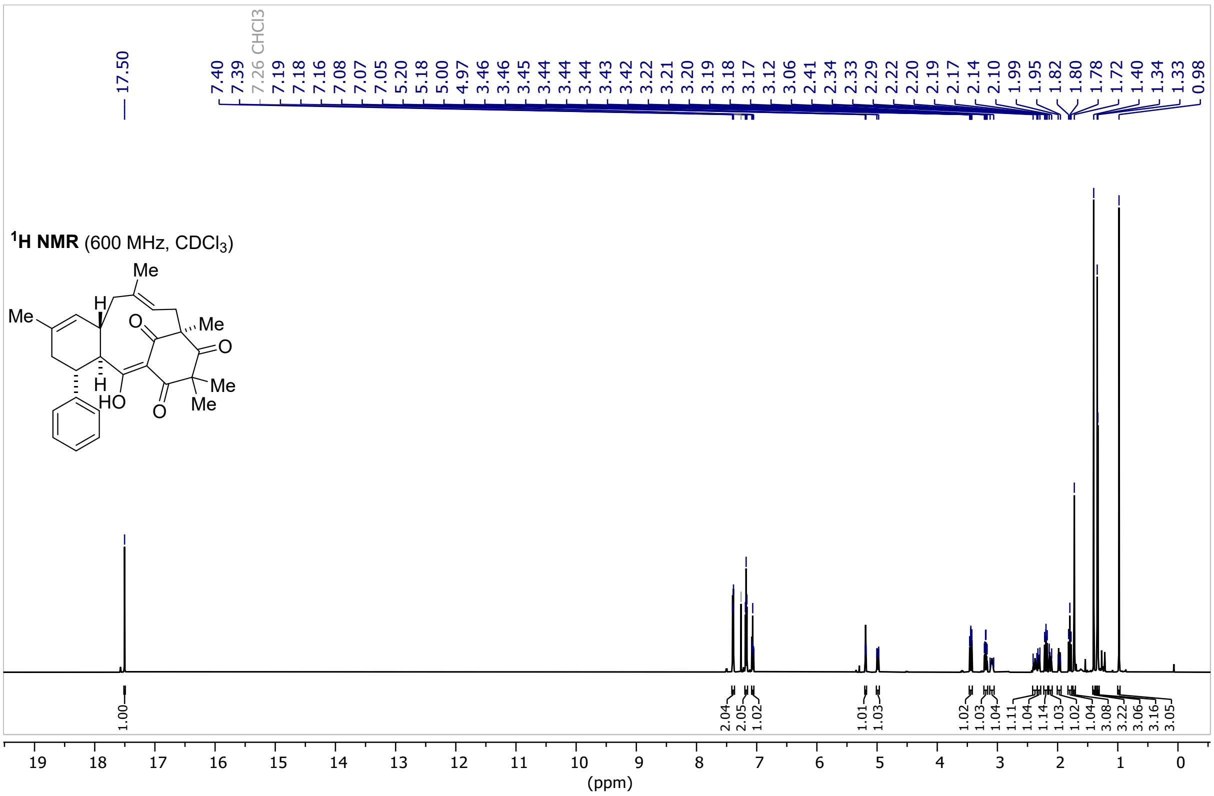

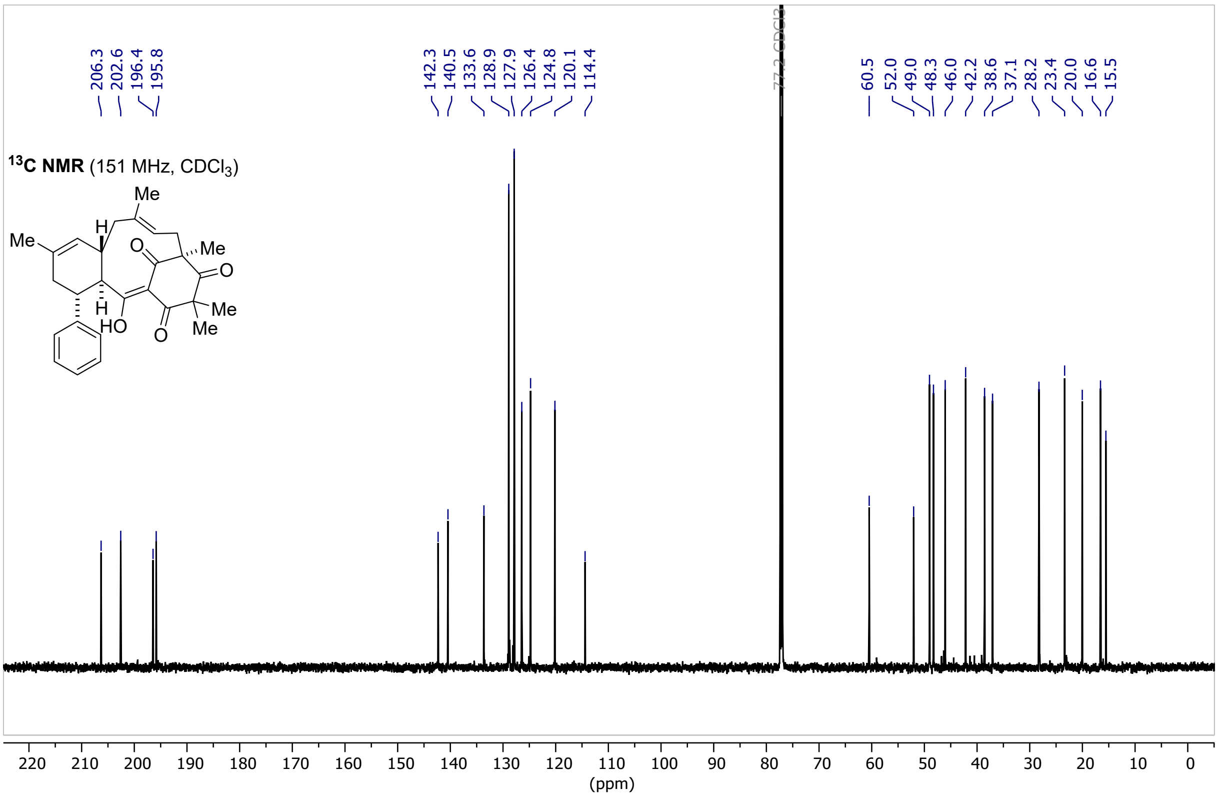

IMDA Diastereomer 8

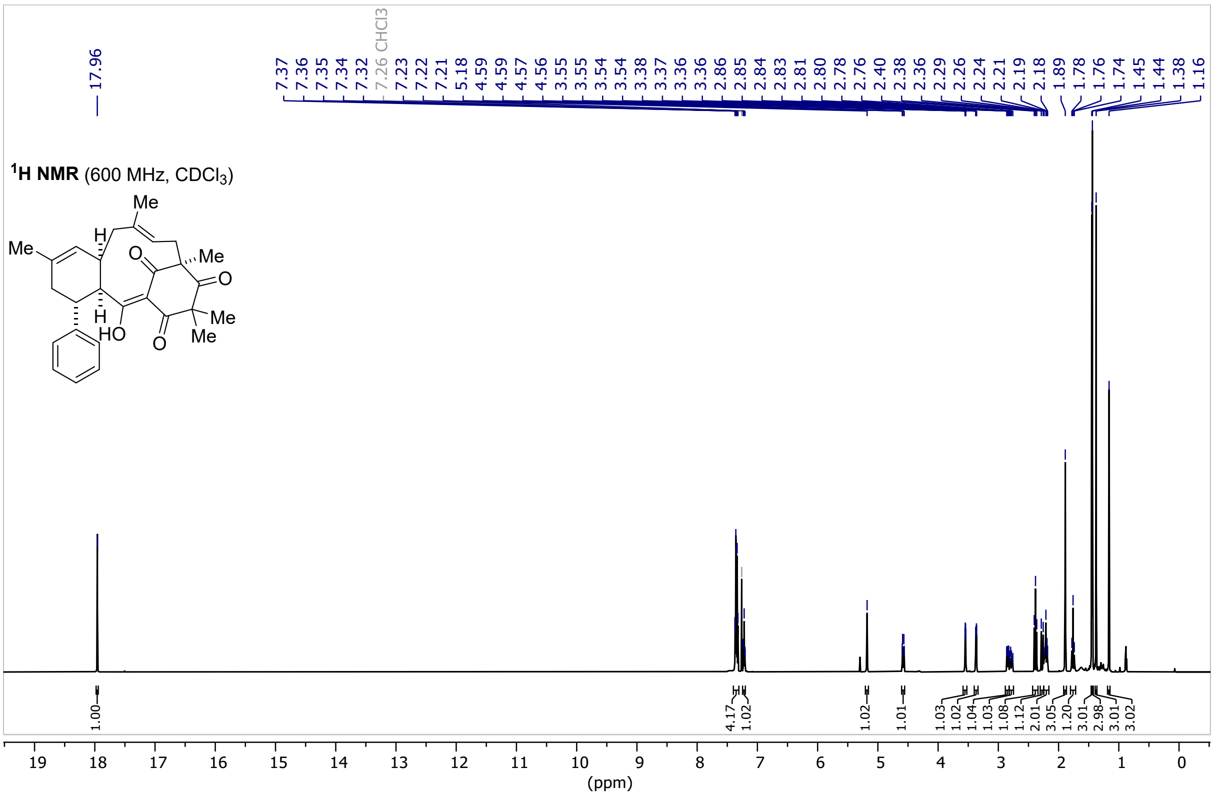

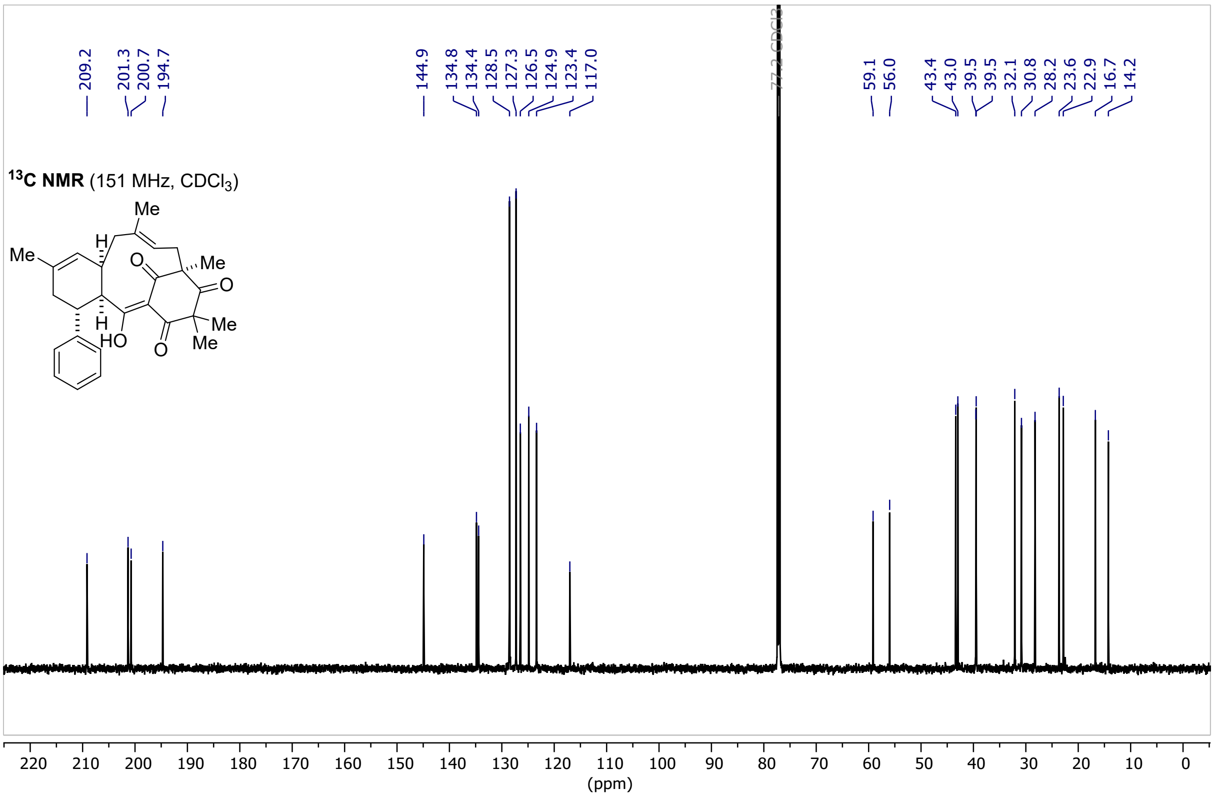

Epoxide 16

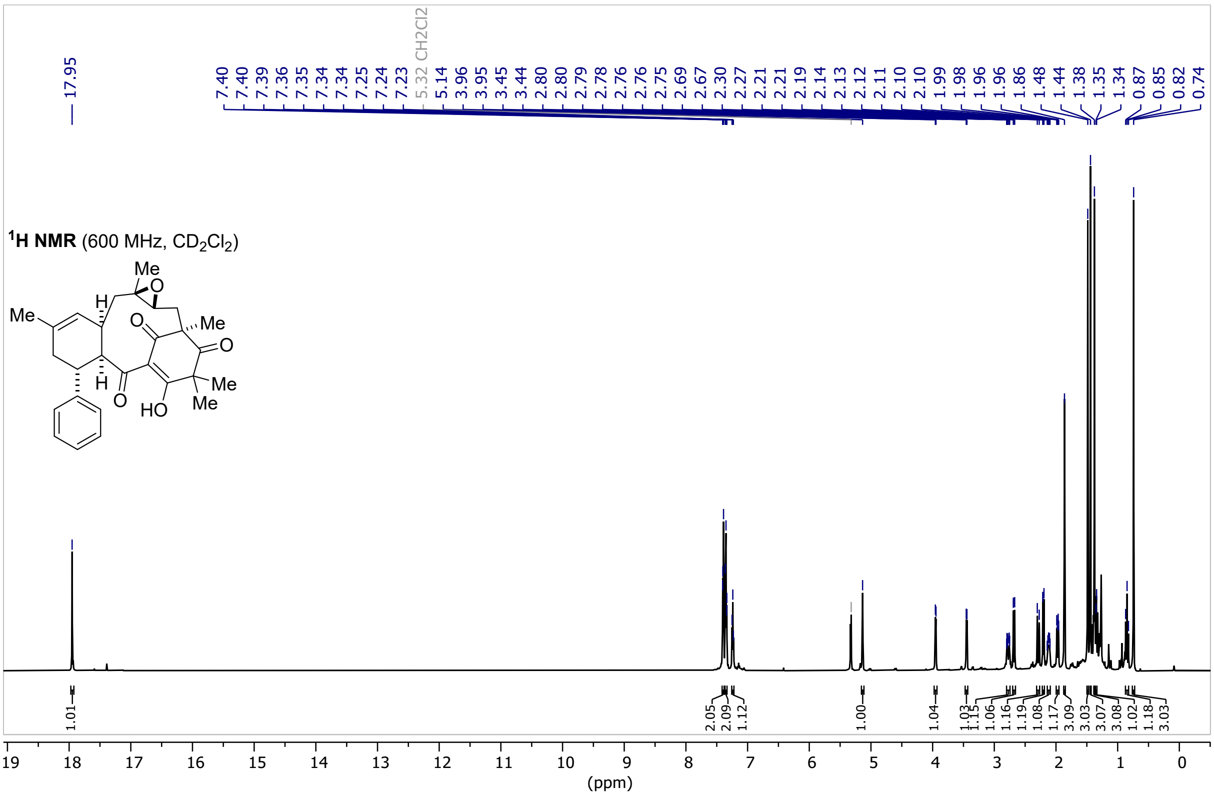

1H NMR (600 MHz, CD2Cl2)
17.95
7.40
7.40
7.39
7.36
7.35
7.34
7.34
7.25
7.24
7.23
5.32 CH2Cl2
5.14
3.96
3.95
3.45
3.44
2.80
2.80
2.79
2.78
2.76
2.76
2.75
2.69
2.67
2.30
2.27
2.21
2.21
2.19
2.14
2.13
2.12
2.11
2.10
2.10
1.99
1.98
1.96
1.96
1.86
1.48
1.44
1.38
1.35
1.34
0.87
0.85
0.82
0.74
(ppm)

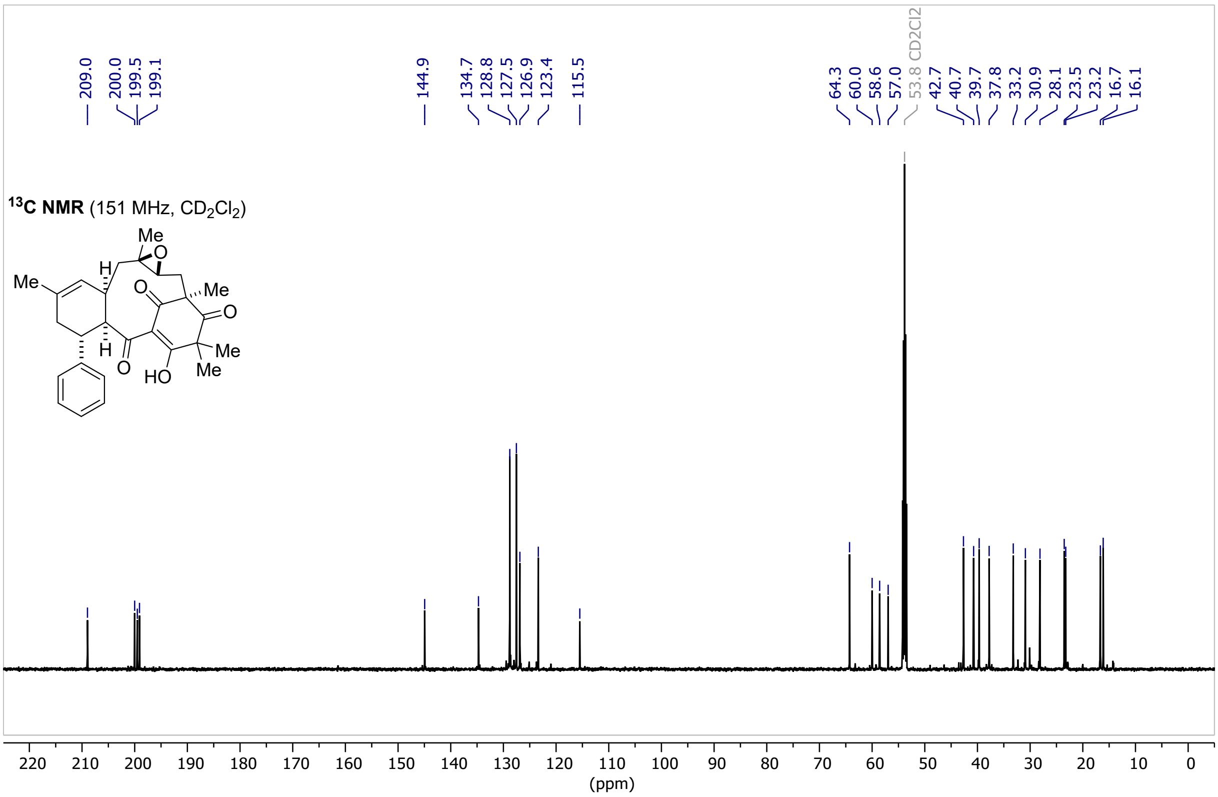

13C NMR (151 MHz, CD2Cl2)
209.0
200.0
199.5
199.1
144.9
134.7
128.8
127.5
126.9
123.4
115.5
64.3
60.0
58.6
57.0
53.8 CD2Cl2
42.7
40.7
39.7
37.8
33.2
30.9
28.1
23.5
23.2
16.7
16.1
(ppm)

(±)-Cleistocaltone A (1)

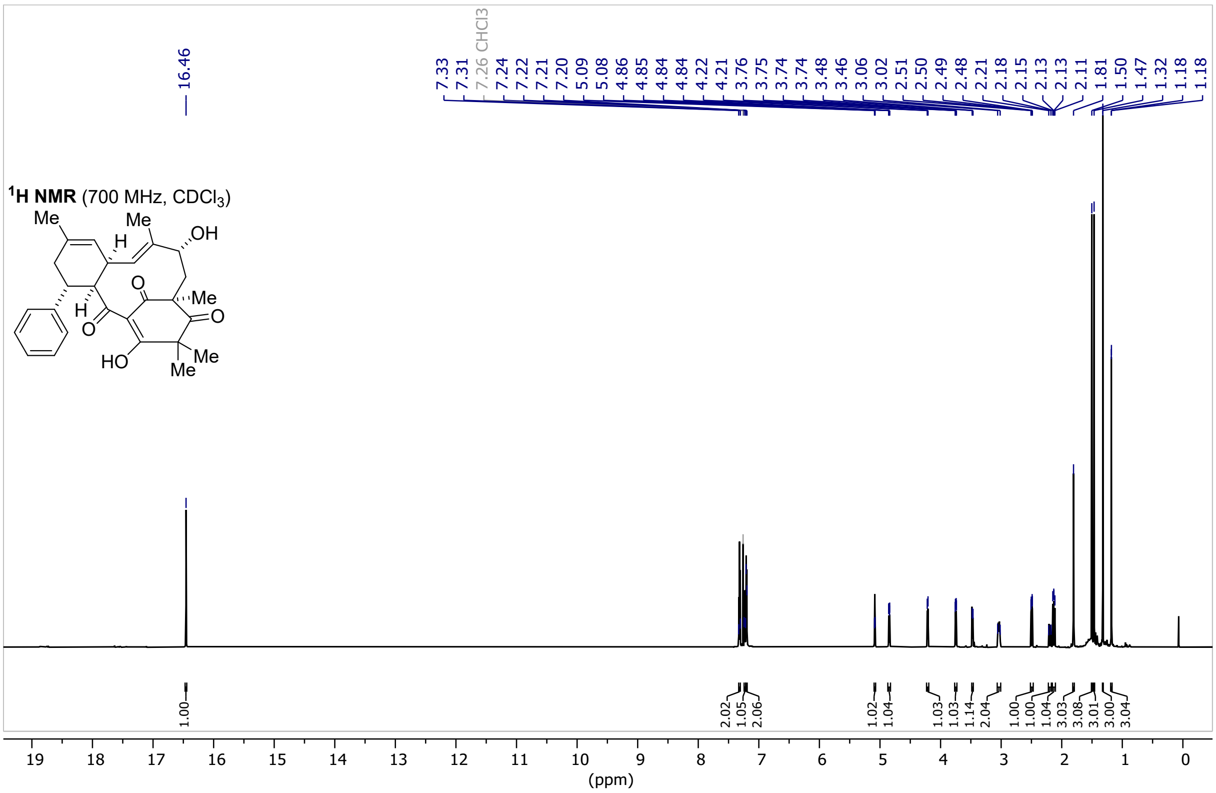

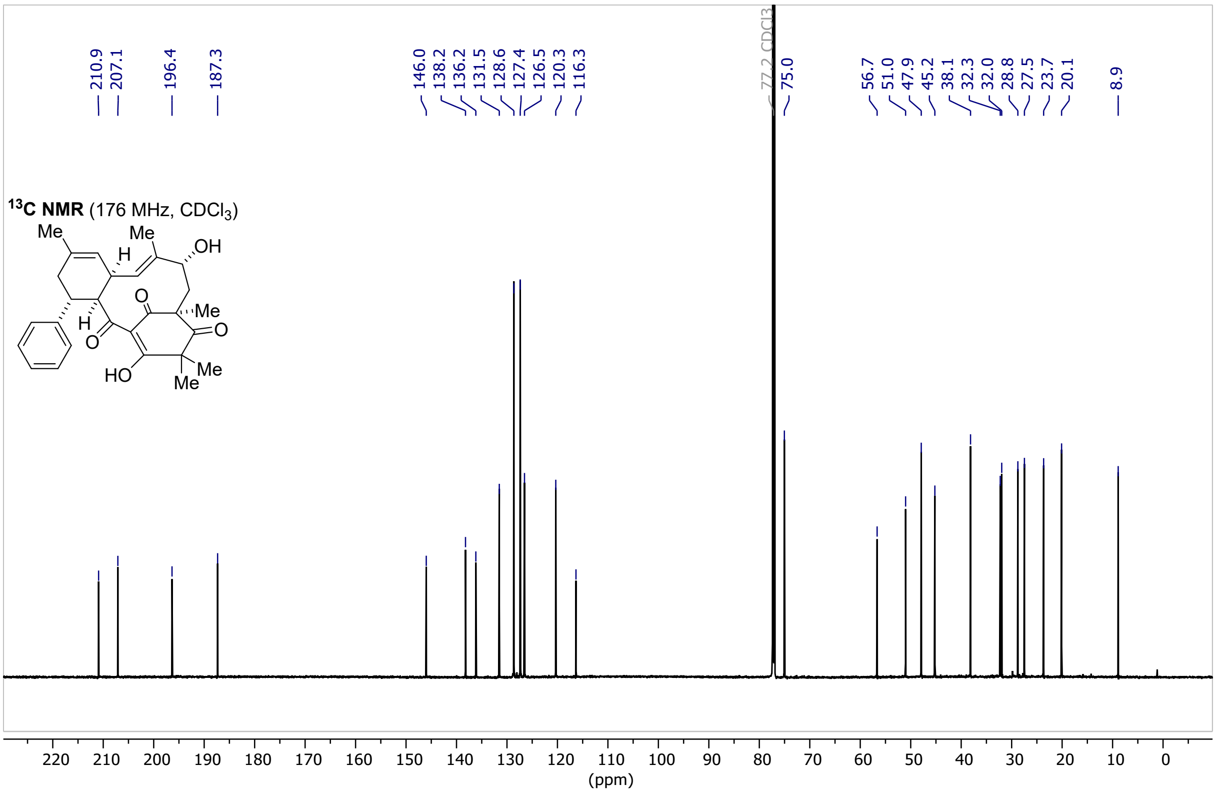

Side Product 17

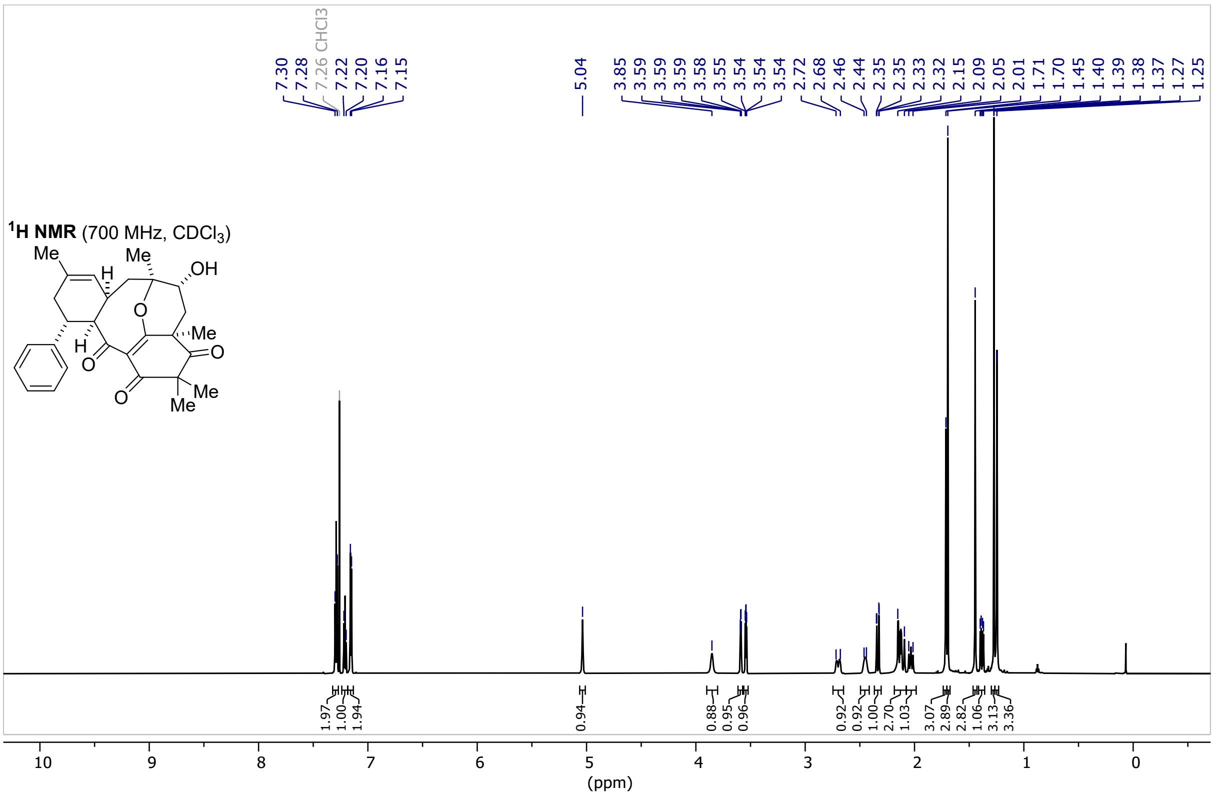

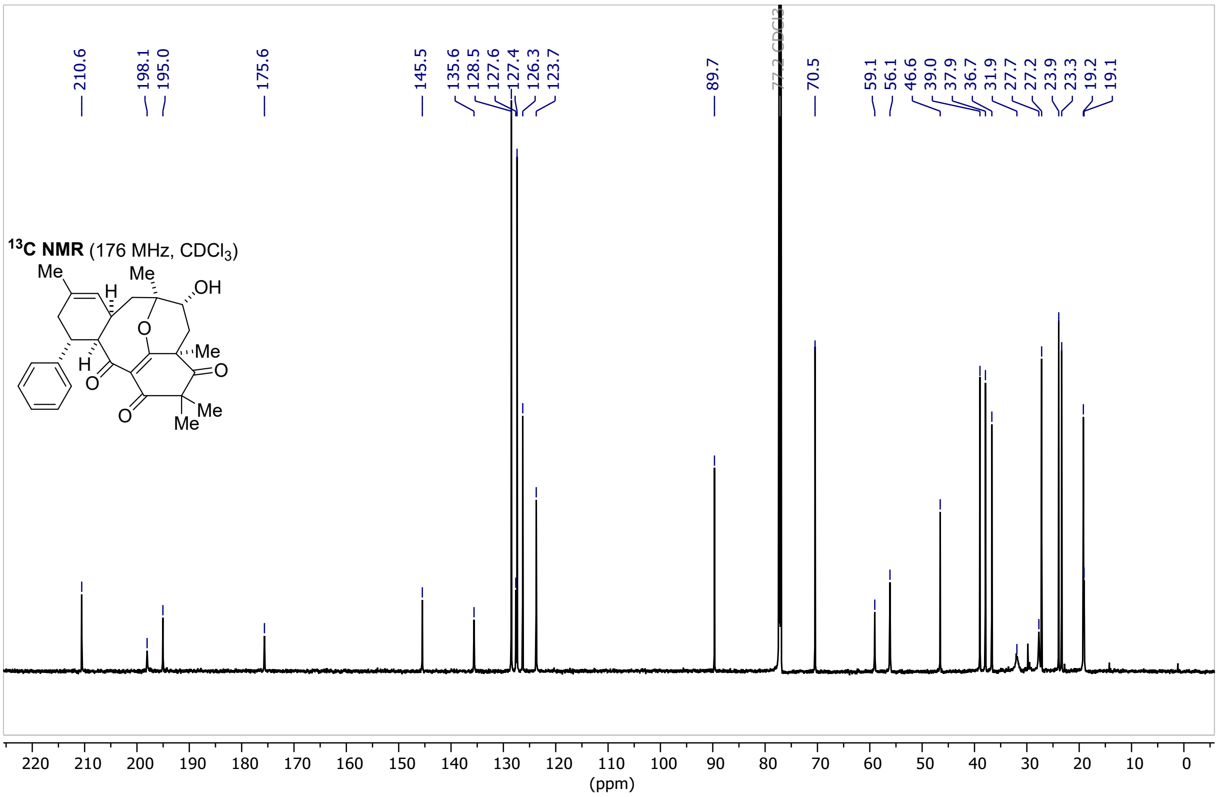

12. References

1 G. M. Sheldrick, *Acta Crystallogr. Sect. A Found. Crystallogr.*, 2008, **64**, 112–122.

2 G. M. Sheldrick, *Acta Crystallogr. Sect. C Struct. Chem.*, 2015, **71**, 3–8.

3 O. V. Dolomanov, L. J. Bourhis, R. J. Gildea, J. A. K. Howard and H. Puschmann, *J. Appl. Crystallogr.*, 2009, **42**, 339–341.

4 W. K. Jo, A. Schadenhofer, A. Habierski, F. K. Kaiser, G. Saletti, T. Ganzenmueller, E. Hage, S. Haid, T. Pietschmann, G. Hansen, T. F. Schulz, G. F. Rimmelzwaan, A. D. M. E. Osterhaus and M. Ludlow, *Proc. Natl. Acad. Sci. U. S. A.*, 2021, **118**, e2026558118.

5 J. P. DeVincenzo, R. J. Whitley, R. L. Mackman, C. Scaglioni-Weinlich, L. Harrison, E. Farrell, S. McBride, R. Lambkin-Williams, R. Jordan, Y. Xin, S. Ramanathan, T. O'Riordan, S. A. Lewis, X. Li, S. L. Toback, S.-L. Lin and J. W. Chien, *N. Engl. J. Med.*, 2014, **371**, 711–722.

6 Solvias AG, Solvias Ligands and Catalysts Library, https://ligands.solvias.com/library/index. (Accessed: 21.03.2024)

7 K. Nakagawa-Goto, J. H. Wu and K. H. Lee, *Synth. Commun.*, 2005, **35**, 1735–1739.

8 J. Q. Hou, C. Guo, J. J. Zhao, Q. W. He, B. B. Zhang and H. Wang, *J. Org. Chem.*, 2017, **82**, 1448–1457.

9 E. Nakamura, S. Aoki, K. Sekiya, H. Oshino and I. Kuwajima, *J. Am. Chem. Soc.*, 1987, **109**, 8056–8066.

10 European Patent Office, EP 1 072 589 A2, 2001, 11–12.

11 J. G. Song, J. C. Su, Q. Y. Song, R. L. Huang, W. Tang, L. J. Hu, X. J. Huang, R. W. Jiang, Y. L. Li, W. C. Ye and Y. Wang, *Org. Lett.*, 2019, **21**, 9579–9583.
